# Colorectal cancer-associated anaerobic bacteria proliferate in tumor spheroids and alter the microenvironment

**DOI:** 10.1038/s41598-020-62139-z

**Published:** 2020-03-24

**Authors:** Stephen H. Kasper, Carolina Morell-Perez, Thomas P. Wyche, Theodore R. Sana, Linda A. Lieberman, Erik C. Hett

**Affiliations:** Exploratory Science Center, Merck & Co., Inc., Cambridge, Massachusetts USA

**Keywords:** Cancer microenvironment, Cellular microbiology

## Abstract

Recent reports show that colorectal tumors contain microbiota that are distinct from those that reside in a ‘normal’ colon environment, and that these microbiota can contribute to cancer progression. *Fusobacterium nucleatum* is the most commonly observed species in the colorectal tumor microenvironment and reportedly influences disease progression through numerous mechanisms. However, a detailed understanding of the role of this organism in cancer progression is limited, in part due to challenges in maintaining *F*. *nucleatum* viability under standard aerobic cell culture conditions. Herein we describe the development of a 3-dimensional (3D) tumor spheroid model that can harbor and promote the growth of anaerobic bacteria. Bacteria-tumor cell interactions and metabolic crosstalk were extensively studied by measuring the kinetics of bacterial growth, cell morphology and lysis, cancer-related gene expression, and metabolomics. We observed that viable *F*. *nucleatum* assembles biofilm-like structures in the tumor spheroid microenvironment, whereas heat-killed *F*. *nucleatum* is internalized and sequestered in the cancer cells. Lastly, we use the model to co-culture 28 *Fusobacterium* clinical isolates and demonstrate that the model successfully supports co-culture with diverse fusobacterial species. This bacteria-spheroid co-culture model enables mechanistic investigation of the role of anaerobic bacteria in the tumor microenvironment.

## Introduction

Colorectal cancer (CRC) is the third most common cancer type and second leading cause of cancer-related deaths in the United States^1^. While genetic predisposition plays a role in some CRCs, many CRCs are caused and/or driven by response to environmental factors^2^. The colon is the most densely populated microbial ecosystem within the human body, and there is mounting evidence for the role of human microbiota in CRC initiation and progression^3–5^. Recent advances in DNA sequencing technologies have resulted in the identification of specific microorganisms that are enriched in the CRC tumor microenvironment (TME).

A frequently identified organism in the CRC TME is *F*. *nucleatum*^6–10^, a Gram-negative anaerobic bacterium, classically associated with oral biofilms and periodontitis^11,12^. However, recent reports have demonstrated a potential role for enhancing cancer cell proliferation^13,14^, modulating tumor immunity^15,16^, regulating autophagy^17^, and influencing metastasis^10,18,19^. Despite these compelling observations, a mechanistic understanding of the role for this organism in cancer progression is limited, in part due to challenges in maintaining the viability of *F*. *nucleatum* under standard aerobic human cell culture conditions. Several studies used conventional 2D cell culture techniques, with particularly high ratios *of F*. *nucleatum*-to-human cells, often up to 1000:1, possibly to account for the lack of bacterial viability and proliferation^13–15,17^.

While these studies have demonstrated important interactions between the surface components of *F*. *nucleatum* and both epithelial and immune cells, they did not reveal any specific effects due to viable *F*. *nucleatum*, or characterize any host-microbe metabolite crosstalk. This is a challenging problem in microbiome research and is beginning to be addressed by the development of engineered models to co-culture host cells with anaerobic bacteria^20–26^. 3D gut organoids, which model the native healthy gut with polarized and differentiated epithelial cells forming a luminal compartment, have also been used to co-culture anaerobic bacteria^27,28^. However, none of these previously reported co-culture models have used *F*. *nucleatum* in a complex environment such as the TME, where this bacterium is found to be enriched. In contrast to 3D gut organoids, 3D tumor spheroids present a unique opportunity to study intra-tumor anaerobic bacteria, as they accurately mimic several solid tumor characteristics, including oxygen and nutrient gradients, as well as heterogeneity in cellular activity (e.g. metabolism, proliferation, cell death)^29–31^. Herein is the first description of a 3D tumor spheroid model co-cultured with cancer-relevant, endogenously found, anaerobic bacteria. Bacteria-spheroid co-cultures (BSCCs) have been previously reported in experiments using genetically tractable anaerobic bacteria as potential gene delivery vectors for therapeutic applications^32,33^. We leveraged the 3D nature of these tumor spheroids to study the effects of co-culturing viable *F*. *nucleatum* with epithelial cells*;* including, gene expression, metabolomics, and their morphology.

## Results

### Microbial viability in a 3D tumor spheroid co-culture model is both species- and spheroid size-dependent

**Figure 1:**
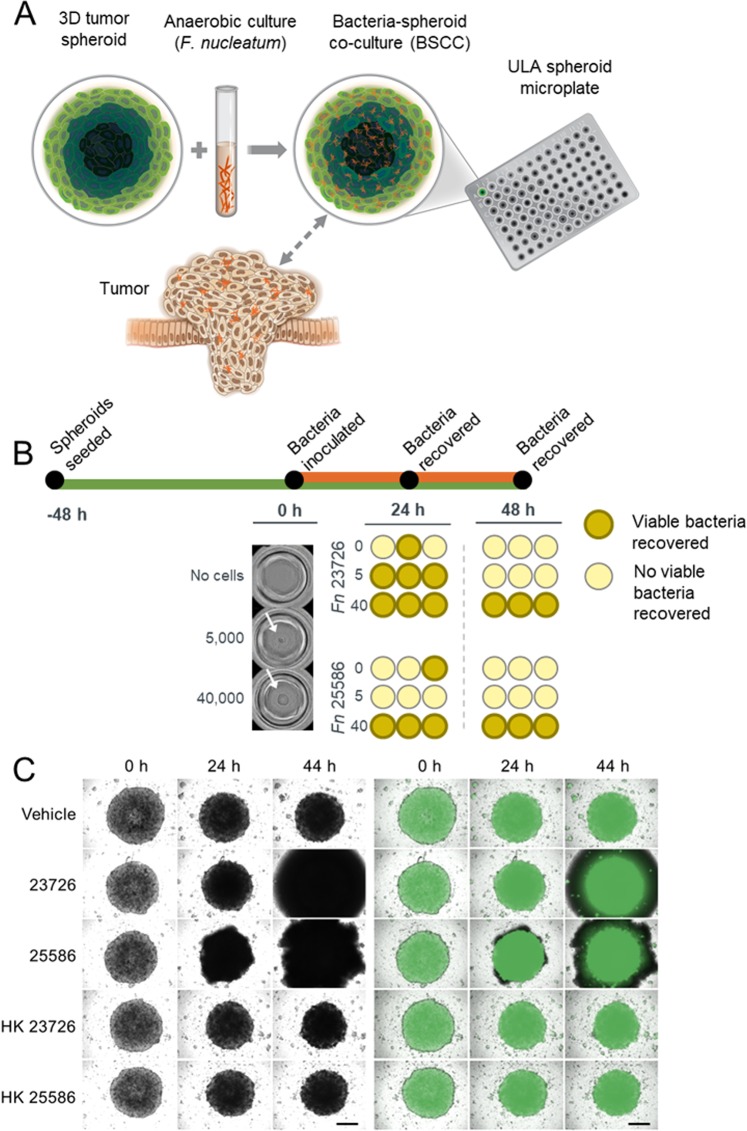
Co-culturing intra-tumor bacteria with colorectal tumor spheroids results in morphological changes to BSCCs. **(A)** Schematic diagram depicting the BSCC model. Microplate-based 3D tumor spheroid technology is used to culture *F*. *nucleatum*. **(B)** Timeline showing the experimental workflow and recovery of viable *F*. *nucleatum*. Tumor spheroids are shown immediately prior to *F*. *nucleatum* inoculation. For scale, tumor spheroids are in a 6.35 mm diameter well (outer most curve). Viable bacteria recovered at 24 h and 48 h are indicated by dark circles with bold outlines. White arrows indicate a single tumor spheroid in the center of the well. **(C)** Bright field (left) and GFP (right) channel images of the BSCC model at 0 h, 24 h and 44 h post infection. From top to bottom showing results for vehicle, *F*. *nucleatum* 23726, *F*. *nucleatum* 25586, HK *F*. *nucleatum* 23726 and HK *F*. *nucleatum* 25586. Scale bar = 300 μm.

Previous observations of tumor spheroids have described characteristic oxygen and nutrient gradients that mimic those in solid tumors. Since *F*. *nucleatum* is consistently found to be enriched in the colorectal TME, we hypothesized that co-culturing anaerobic *F*. *nucleatum* with tumor spheroids may provide a niche for maintaining *F*. *nucleatum* viability outside the anaerobic chamber (Fig. 1A). To test this hypothesis, we developed a BSCC model. Two commonly used laboratory strains of *F*. *nucleatum*, ATCC 23726 and ATCC 25586, were co-cultured with varying sizes of tumor spheroids from the human colorectal adenocarcinoma cell line HT-29. Following incubation under aerobic conditions for 24 h or 48 h, the BSCCs were transferred into bacterial broth in an anaerobic environment to determine if bacteria remained viable. After 24 h, *F*. *nucleatum* 23726 was recovered from 1/3 wells with no tumor spheroids present, and from 3/3 wells with either a 5,000 cell or 40,000 cell tumor spheroid present (Fig. 1B). Similarly, *F*. *nucleatum* 25586 was recovered from 1/3 wells without a tumor spheroid, from 0/3 wells with 5,000 cell tumor spheroids, and from 3/3 wells when co-cultured with 40,000 cell tumor spheroids (Fig. 1B). When the same experiment was carried out over 48 h, neither strain was recovered in a viable condition from wells without tumor spheroids, or from wells containing 5,000 cell tumor spheroids. However, we were able to recover both strains from 3/3 wells when starting with 40,000 cell tumor spheroids (Fig. 1B). Similar results were observed for intermediate sized tumor spheroids (i.e. 10,000 or 20,000 cells) and for another human colon cancer cell line, HCT 116 (Supplementary Fig. 1). When testing the BSCC model with a different anaerobic species, *Faecalibacterium prausnitzii*, which is not commonly associated with CRC^7,34^, no viable bacteria could be recovered at any timepoint for any tumor spheroid size (Supplementary Fig. 1), suggesting that this niche environment cannot universally support the growth and viability of all anaerobic microbes. Based on these results, we determined that BSCCs can harbor viable *F*. *nucleatum* for at least 48 h and that 40,000 cell BSCCs consistently yielded viable *F*. *nucleatum*.

We used live cell imaging to determine if any morphological changes could be observed in BSCCs. At 24 h after inoculation, a halo of biomass (based on bright-field imaging) began to emerge from BSCCs with *F*. *nucleatum* (Fig. 1C). The biomass halo was not observed in BSCCs with either heat-killed (HK) *F*. *nucleatum* strains, or when smaller tumor spheroid sizes (<40,000 cells; Fig. 1C, Supplementary Fig. 1) were used. When a GFP-labeled HT-29 cell line was used, the biomass halo did not show a fluorescent signal, suggesting that the halo was not composed of tumor spheroid cells, and was instead likely to be a bacteria emerging from the inside or underneath the BSCC structure (Fig. 1C).

### Bacterial growth is associated with increased cytotoxicity to tumor spheroids

**Figure 2:**
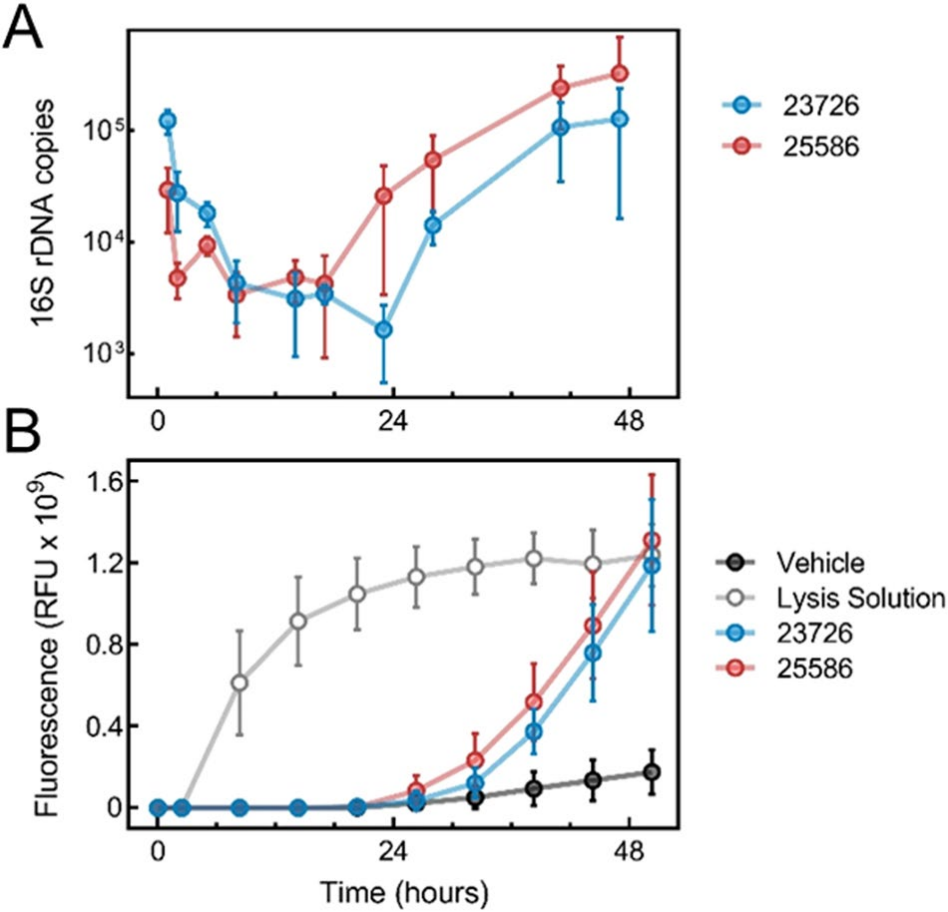
Bacterial growth and tumor spheroid viability kinetics in BSCCs. **(A)** *F*. *nucleatum* 16s rRNA gene qPCR from the BSCC model over a 48 h period. Results are shown as 16s rDNA copies over time for BSCCs with *F*. *nucleatum* strains 23726 (blue) and 25586 (red). **(B)** Tumor spheroid cytotoxicity over 48 h after inoculation with *F*. *nucleatum*. Increased fluorescence is indicative of increased cytotoxicity in the dye exclusion assay. Results show BSCCs with *F*. *nucleatum* 23726 (blue), 25586 (red), lysis solution treatment (white), or vehicle control (black). Data represent the mean and standard deviation of at least three biological replicates.

We sought to quantify the *F*. *nucleatum* bacterial load in BSCCs over time. We isolated total DNA from BSCCs and used qPCR amplification of the 16S rRNA gene to quantify *F*. *nucleatum* abundance, as compared to CFU counting, which would have relied on efficiently dismantling BSCCs without affecting *F*. *nucleatum* viability. We observed a rapid decrease in 16S rRNA gene abundance that reached a minimum around 12 h in both strains of *F*. *nucleatum*, indicating bacterial death likely due to oxygen exposure in the aerobic environment (Fig. 2A). However, around 24 h, we observed a logarithmic increase in 16S rRNA gene abundance (Fig. 2A), suggesting logarithmic growth of both *F*. *nucleatum* strains in the BSCC model. When cultured in the same media without the tumor spheroids, both *F*. *nucleatum* strains showed moderate growth in an anaerobic environment, and no growth in an aerobic environment (Supplementary Fig. 2).

We tracked the fluorescent signal of GFP-labeled BSCCs over time and observed that the GFP signal became more diffuse (Fig. 1C) at later time points when incubated with viable *F*. *nucleatum*. We also noticed that upon pipetting, BSCCs were more fragile, indicating that *F*. *nucleatum* could be cytotoxic to the tumor spheroids. To characterize this effect over time, we employed a fluorescent dye exclusion viability assay. Through the first ~24 h, virtually no HT29 cytotoxicity was detected (Fig. 2B). However, between 24–48 h, during the logarithmic growth phase, a steep increase in cytotoxicity was detected in BSCCs containing either one of the two viable *F*. *nucleatum* strains (Fig. 2B). These results suggest that uncontrolled *F*. *nucleatum* growth could have cytotoxic effects toward colorectal cancer cells.

### Viable *F*. *nucleatum* assemble extracellular biofilm-like aggregates in tumor spheroid microenvironment whereas HK bacteria are internalized

**Figure 3:**
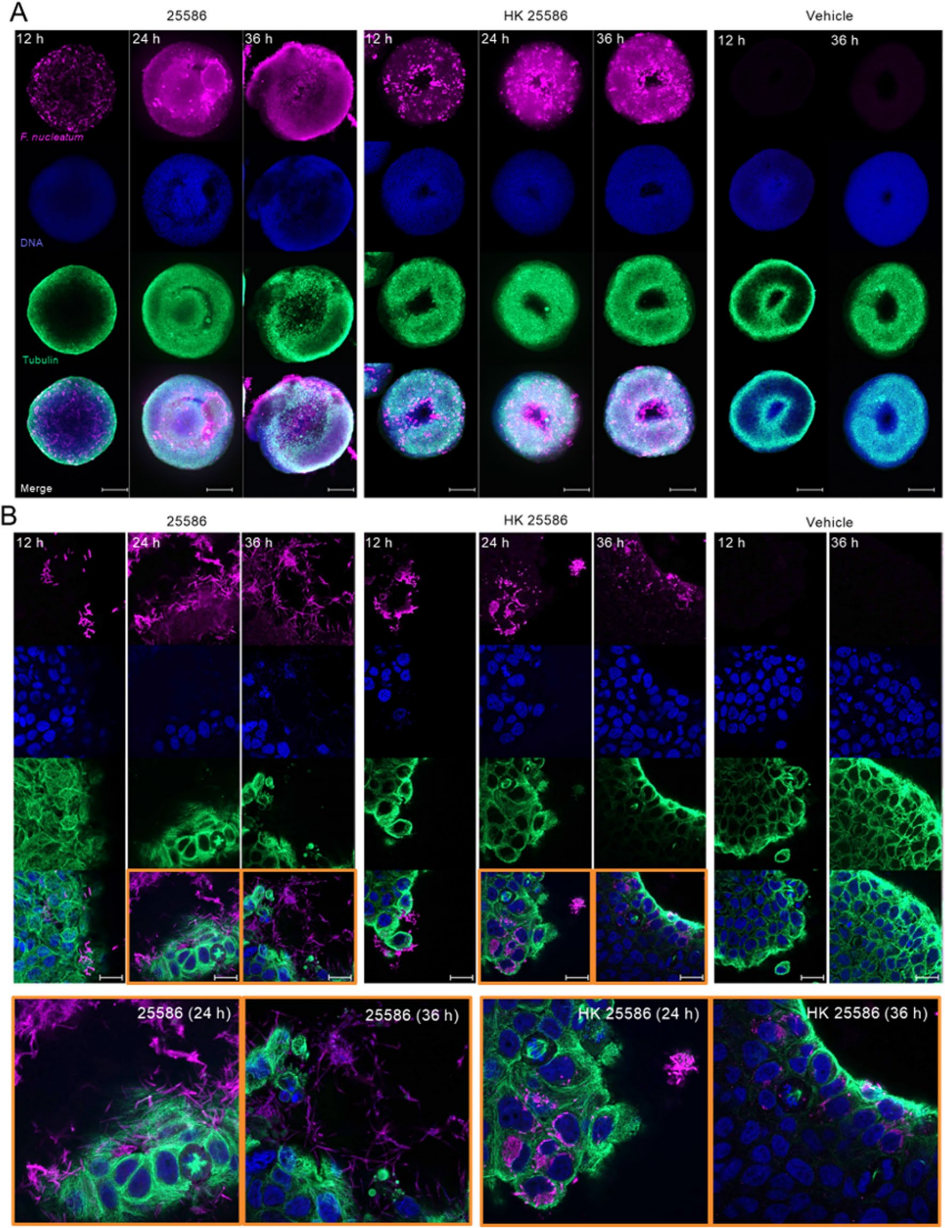
Confocal laser scanning microscopy of BSCCs. **(A)** Low magnification (5×) images of BSCCs with viable *F*. *nucleatum*, HK *F*. *nucleatum*, or vehicle control. Scale bar = 200 μm. **(B)** High magnification (63×) images of the same conditions shown above. Scale bar = 20 μm. Enlarged images of BSCCs with viable and HK *F*. *nuclea*tum at 24 h and 36 h are indicated in orange outline. For both panels, single channel acquisition for anti-*F*. *nucleatum* 25586 is shown in the top row in fuchsia, Hoechst stain showing cell nuclei in the second row in blue, anti-tubulin is shown in the third row in green, and the merge is shown in the bottom row.

To gain additional insights into the arrangement of *F*. *nucleatum* in the tumor spheroid microenvironment, we used fluorescent immunocytochemical staining and confocal laser scanning microscopy (CLSM) on BSCCs with viable *F*. *nucleatum*, HK *F*. *nucleatum*, or vehicle control at 12 h, 24 h, and 36 h timepoints. At low magnification (5×), BSCCs displayed a hollow core, perhaps resulting from a zone of necrotic cells, previously reported to occur in larger spheroids (>500 μm)^30,31,35^. Aggregates of bacteria with relatively even distribution throughout the BSCCs were observed at 12 h, when they were co-cultured with viable or HK *F*. *nucleatum* 25586. However, viable *F*. *nucleatum* aggregates appeared to be smaller and more evenly distributed (Fig. 3A). At 24 h, viable *F*. *nucleatum* was more diffuse, with a few visible aggregates and a large *F*. *nucleatum*-based biomass emerging from near the BSCC center (Fig. 3A). In contrast to BSCCs with viable bacteria, BSCCs with HK *F*. *nucleatum* displayed dozens of visible aggregates at 24 h (Fig. 3A). This suggested that viable and HK *F*. *nucleatum* were differentially distributed in the tumor spheroid microenvironment. A distinct center mass of cancer cells was observed in BSCCs with viable *F*. *nucleatum* that was not apparent in vehicle- or HK-treated BSCCs (Fig. 3A). At 36 h, the viable *F*. *nucleatum*-based biomass was observed to be protruding from the BSCC, with significant HT29 cytoskeletal rearrangement, whereas HK *F*. *nucleatum*-treated BSCCs looked largely unchanged from the 24 h timepoint (Fig. 3A).

BSCCs were also observed under high magnification (63×). At 12 h, both the viable and HK *F*. *nucleatum* appeared to be in direct contact with the cancer cells (Fig. 3B), and to be actively engaged with the cytoskeleton, as evidenced by colocalization with tubulin staining (Fig. 3B). By 24 h, a bacterial biomass had accumulated in the viable *F*. *nucleatum* co-culture, forming a structure resembling a biofilm, while the HK aggregates remained similar in size (Fig. 3B). At 24 h, we also observed that the tubulin structure of BSCCs had been compromised when compared to vehicle control or to the 12 h viable *F*. *nucleatum* condition (Fig. 3B). On the other hand, for the 24 h HK condition, we observed an intact cytoskeleton with cellular internalization of the bacteria taking place (Fig. 3B). By 36 h, viable *F*. *nucleatum* displayed filamentous growth when compared to the same condition at 12 h. The observations from fluorescence CLSM further support our earlier conclusion that viable *F*. *nucleatum* forms biofilm-like biomass and progressively damages the structural integrity of tumor spheroids at 24 h and beyond, which was not observed in HK *F*. *nucleatum* or in vehicle conditions.

### Viable *F*. *nucleatum* differentially regulates cancer-related gene expression under proliferating conditions

**Figure 4:**
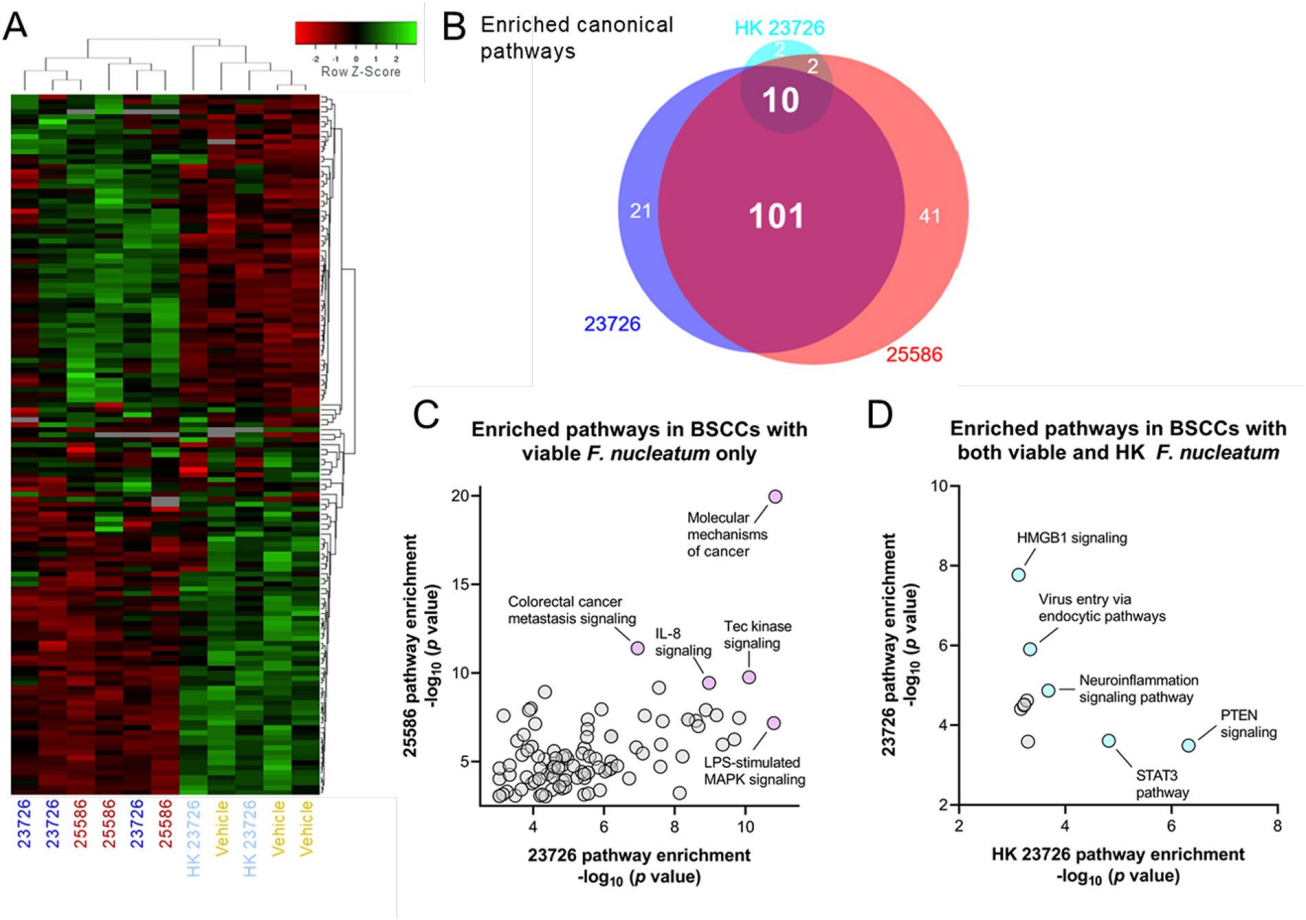
Transcriptomic analysis of BSCCs using a high-throughput qPCR cancer gene expression panel. **(A)** Hierarchical clustering of differentially expressed genes in HT29 cells based on dCt values. **(B)** Venn diagram for the number of enriched and overlapping canonical pathways in BSCCs with *F*. *nucleatum* 23726, 25586, or HK *F*. *nucleatum* 23726. **(C)** Scatter plot of enriched canonical pathways that are shared between BSCCs with viable *F*. *nucleatum* (101 total, as indicated in (**B**)). **(D)** Scatter plot of enriched canonical pathways that are shared between BSCCs with viable and HK *F*. *nucleatum* (10 total, as indicated in (**B**)). Pathways are plotted by p-value (Fisher’s exact test) for each condition.

*F*. *nucleatum* has been consistently associated with CRCs and detected in tumor tissues^6–9,14,19,36–38^, and high levels of *Fusobacteria* are reported to be associated with a worsening prognosis in cancer patients^39^. Therefore, in an effort to understand how viable *F*. *nucleatum* affects gene expression changes in cancer cells, we used high-throughput qPCR to measure the expression of >500 cancer-related genes. BSCCs were co-cultured with viable or HK *F*. *nucleatum* 23726, viable *F*. *nucleatum* 25586, or vehicle control (medium alone) for 24 h before RNA was isolated and subjected to qPCR. Pairwise analyses of all three conditions versus vehicle control resulted in a combined total of 137 significantly differential expressed genes (DEGs; *p* < 0.05, unpaired T-test). BSCCs with *F*. *nucleatum* 25586 yielded 111 DEGs; *F*. *nucleatum* 23726 yielded 68 DEGs; and HK *F*. *nucleatum* 23726 yielded 18 DEGs, with considerable DEG overlap between the treatments (Supplementary Table 2, Supplementary Fig. 3). DEGs were visualized in heat maps after hierarchical cluster analysis using Average Linkage (Pearson distance measurement, Fig. 4A) for individual replicates. The replicate measurements for viable *F*. *nucleatum* clustered together and separately from HK *F*. *nucleatum* and vehicle controls (Fig. 4A).

Canonical pathway enrichment of DEGs suggested that 177 different pathways were significantly enriched (*p* < 0.001, Fisher’s exact test) between all three conditions; of which 101 were common between the BSCCs with the two viable strains and 10 in common between viable and HK conditions (Fig. 4B). Several of the most highly enriched pathways in BSCCs with viable *F*. *nucleatum*, but not in BSCCs with HK *F*. *nucleatum*, included molecular mechanisms of cancer, colorectal cancer metastasis signaling, and IL-8 signaling (Fig. 4C). We measured IL-8 protein levels at 24 h and observed ~2–3 fold greater IL-8 in BSCCs with viable *F*. *nucleatum* compared to vehicle or the respective HK strain (Supplementary Fig. 4). Canonical pathways that were most highly enriched in BSCCs with both viable *F*. *nucleatum* and HK *F*. *nucleatum*, include PTEN signaling, STAT3 pathway, and virus entry via endocytic pathways (Fig. 4D). These pathways connect with findings in the literature and are further detailed in the discussion section.

When functional annotations of related DEGs were analyzed, there were increased numbers of disease functions attributed to co-culturing with viable *F*. *nucleatum* compared to HK *F*. *nucleatum* 23726 (Supplementary Fig. 4). Whereas the predicted activation state of “apoptosis in colorectal cancer cell lines” was negative for HK *F*. *nucleatum* 23726 treatment (Supplementary Fig. 4), it was positive in BSCCs with viable *F*. *nucleatum* (Supplementary Fig. 4). Altogether, these results support increased and differential biological activity at the transcriptional level when *F*. *nucleatum* is viable/proliferating in the tumor spheroid microenvironment.

### Viable *F*. *nucleatum* affects metabolite levels in tumor spheroid microenvironment

**Figure 5:**
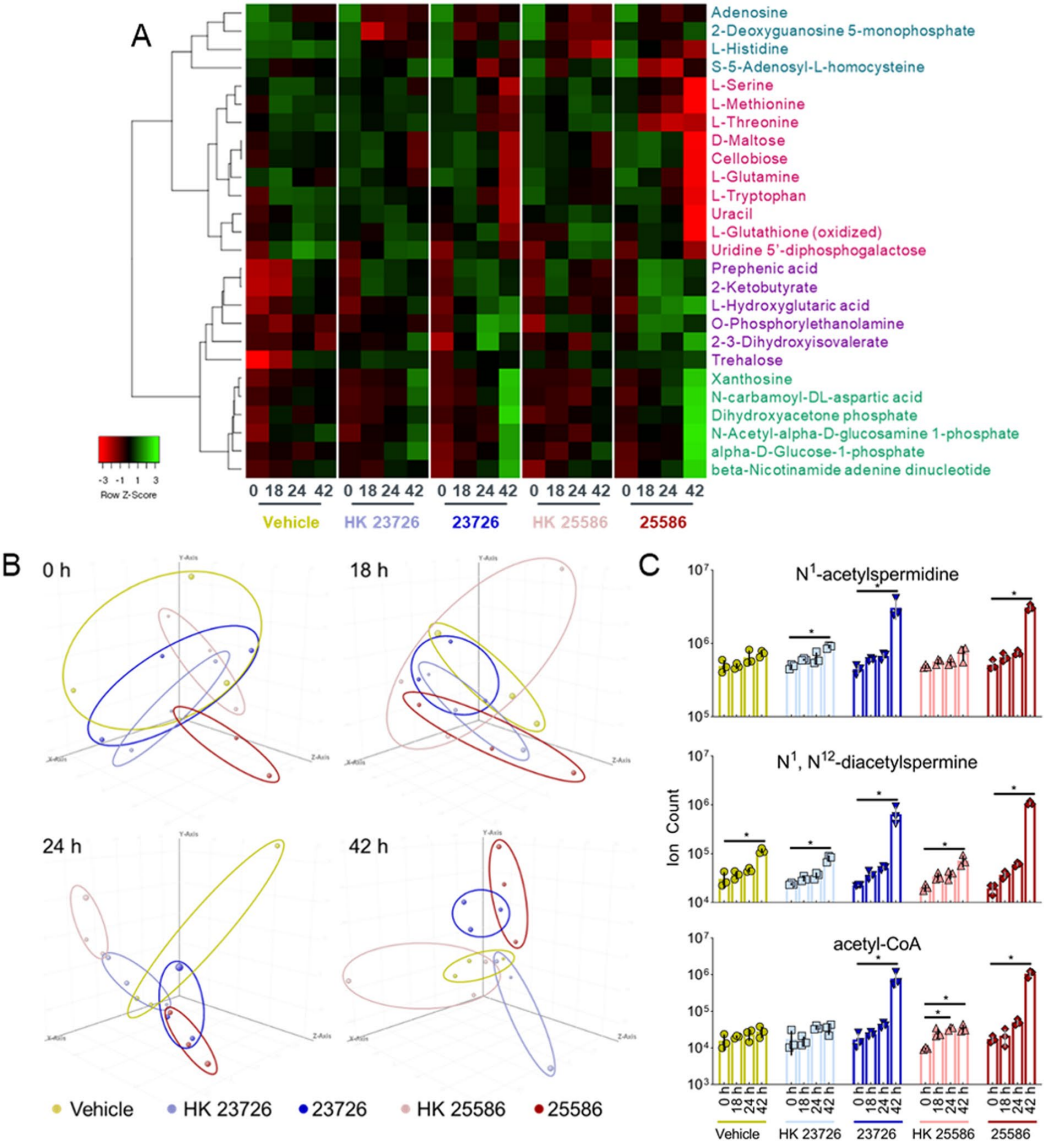
Longitudinal metabolomic analysis of BSCCs. **(A)** Hierarchical clustering of differentially abundant metabolites (*p* < 0.05) in BSCCs. Time (in hours) after BSCC inoculation is shown for each treatment below heat map. **(B)** PCA of the global metabolome detected using Q-TOF LC/MS. Each plot represents a timepoint after BSCC inoculation. **(C)** Mass spectral abundance of several metabolites in BSCCs at four timepoints (*p < 0.01, one-way ANOVA with Dunnett’s multiple comparisons test).

We used a combination of targeted and untargeted metabolomics to determine if viable *F*. *nucleatum* alters metabolic processes in the tumor spheroid microenvironment. Targeted triple quadrupole LC-MS analysis of 155 central carbon metabolites (Supplementary Table 5) of BSCCs with viable *F*. *nucleatum*, HK *F*. *nucleatum*, or vehicle were measured at 0 h, 18 h, 24 h, and 42 h after inoculation. Cell-associated metabolite levels of BSCCs with viable or HK *F*. *nucleatum* were compared to vehicle-treated control BSCCs at each timepoint. Significantly different metabolites (*p* < 0.05, one way ANOVA) between any combination of treatments or timepoints were visualized in a heat map after hierarchical cluster analysis using Average Linkage (Pearson distance measurement, Fig. 5A) analysis. The BSCCs with viable *F*. *nucleatum* had metabolite patterns that distinguished them from BSCCs with heat-killed *F*. *nucleatum* or vehicle, most notably at the 42 h timepoint (Fig. 5A, Supplementary Fig. 5). Previous reports have described amino acids as the preferred substrate for energy generation in *F*. *nucleatum*^40,41^. Our results revealed that the levels of six different amino acids (histidine, tryptophan, glutamine, serine, methionine, threonine) decreased over time in BSCCs with viable *F*. *nucleatum*. Also, no significant accumulation of any amino acids was observed in these BSCCs (Fig. 5A, Supplementary Fig. 5).

Untargeted, global metabolomics analysis of the same samples and timepoints revealed 1,707 unique *m/z*-RT pairs across all samples and conditions. Principal component analysis (PCA) plots were generated for each time point (Fig. 5B). At 0 h and 18 h, considerable overlap between BSCCs with viable *F*. *nucleatum*, HK *F*. *nucleatum*, and vehicle was observed, suggesting that at relatively early timepoints the metabolite levels for these conditions did not vary significantly (Fig. 5B). However, at 24 h, the PCA plots show that the metabolite levels for BSCCs cultured with viable *F*. *nucleatum*, begin to separate from that of BSCCs with HK *F*. *nucleatum* or vehicle-treatment. At 42 h, the different groups of viable *F*. *nucleatum* and HK-/vehicle-treated BSCCs clearly separate (Fig. 5B). This suggests that throughout the course of the co-culture experiment, viable *F*. *nucleatum* affects the tumor spheroid microenvironment and shapes the global metabolome. These results also demonstrate that the BSCC model is capable of capturing metabolic responses of tumor spheroids to the presence of viable and proliferating CRC-relevant bacteria.

In a previous report, patient-derived colorectal tumors that were biofilm-positive had higher levels of polyamines compared to biofilm-negative, with N^1^,N^12^-diacetylspermine being the most significant^42^. Using the data generated from our high resolution, accurate mass spectrometer, we identified N^1^,N^12^-diacetylspermine and N^1^-acetylspermidine, confirmed by injection of authentic standards. The abundances for these metabolites slowly increased over time in BSCCs with HK *F*. *nucleatum* or vehicle-treatment, but drastically increased in BSCCs with viable *F*. *nucleatum* between 24 h and 42 h (Fig. 5C). This suggests that metabolic alterations detected in patient-derived tumors can be recapitulated in the BSCC model.

In addition to detecting dozens of other metabolites, we also identified acetyl-CoA (Fig. 5C). Acetyl-CoA is utilized by spermidine/spermine N^1^-acetyltransferase (SSAT) in the production of acetylated polyamines. Acetyl-CoA can also be used for fermentation by *F*. *nucleatum*, yielding butyrate, a preferred energy source for colonocytes that may have varying roles in CRC development^40,43^.

### Diverse clinical isolates of *Fusobacterium* remain viable in BSCCs and affect BSCC morphology

**Figure 6:**
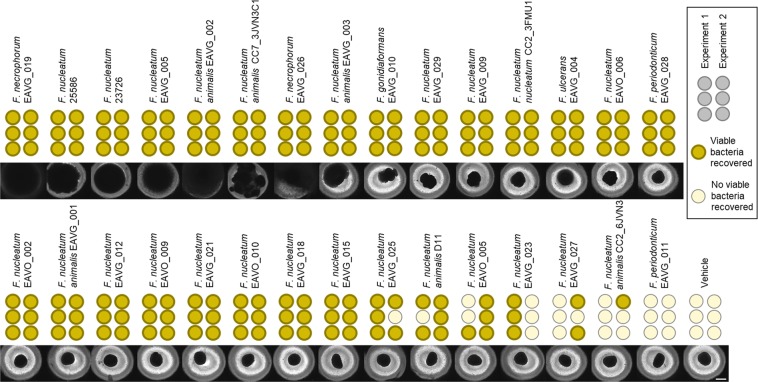
BSCCs with 28 *Fusobacterium* isolates, along with *F*. *nucleatum* 23726 and *F*. *nucleatum* 25586, imaged 48 h after infection. Viable bacteria recovered from two separate experiments are indicated by dark circles with bold outline. BSCCs are ranked, left to right, by morphological effect on BSCCs and frequency of recovery. Scale bar = 500 μm.

While *F*. *nucleatum* 23726 and *F*. *nucleatum* 25586 are model strains that are often used for *in vitro* CRC research^14,15,17,44^, we sought to determine if a greater diversity of *Fusobacteria* can persist in the BSCC model^45^. Therefore, we co-cultured HT29 tumor spheroids with an additional 28 clinical isolates of *Fusobacterium*, mostly consisting of *F*. *nucleatum*, but also comprised of *Fusobacterium gonidiaformans*, *Fusobacterium necrophorum* (2 strains), *Fusobacterium periodonticum* (2 strains), and *Fusobacterium ulcerans*. After 48 h of co-culture, BSCCs were imaged and then transferred into bacterial broth in an anaerobic environment to determine if bacteria remained viable. In addition to *F*. *nucleatum* 23726 and 25586, 21 of the clinical isolates were consistently viable (Fig. 6). Each of the five *Fusobacterium* species tested were represented within this group. As seen with *F*. *nucleatum* 23726 and 25586, several of these strains displayed the notable biofilm-like growth (Fig. 6; left to right, EAVG_019 through EAVG_003). This phenotype appeared to be limited to *F*. *nucleatum* and *F*. *necrophorum* strains in the BSCC model. Other strains induced irregular BSCC morphological changes, including bacterial growth outward of the BSCC (Fig. 6; EAVG_010, through EAVG_028), but not the same extent of biofilm-like growth as the aforementioned 8 strains. Other BSCCs with viable *Fusobacteria* showed no obvious morphological changes through 48 h (Fig. 6; EAVO_002 through EAVG_015). Several strains had intermediate viability (or varying frequency of successful recovery), all of which had no obvious effect on BSCC morphology (Fig. 6; EAVG_025 through CC2_6JVN3). We were unable to recover only one strain, *F*. *periodonticum* EAVG_011, from the BSCC model (Fig. 6).

To determine how the tumor spheroid might influence *Fusobacteria* growth morphology, *Fusobacteria* were similarly seeded into the same media and microplates (excluding tumor spheroids) and incubated for 48 h in anaerobic conditions. From this, *Fusobacteria* formed diverse aggregates and microcolonies that showed no obvious relation to their growth characteristics in the BSCC model (Supplementary Fig. 5). This observation demonstrates that these bacteria are phenotypically different when grown in the BSCC model versus when grown under similar conditions without tumor spheroids in an anaerobic chamber.

## Discussion

*In vitro* models are important tools that can be applied towards gaining mechanistic understanding, rapid hypothesis testing, and screening for tool compounds or therapeutic candidates. With the advent of new sequencing technologies, our appreciation for the role of the microbiome in various disease scenarios is rapidly expanding; however, previous attempts at building *in vitro* co-culture models to recapitulate these interactions have not evolved at the same pace. While the enrichment of *Fusobacteria* in the TME of CRC patients is a common finding^6–9,19,38,39^, and viable *Fusobacteria* can be cultured from the tumor^10^, there is currently a gap in our understanding of how the viability of these bacteria affect the TME. 3D cell culture technologies have the potential to bridge these knowledge gaps. 3D gut organoids are physiologically relevant systems that can be used to characterize interactions of various microbiota with a healthy, differentiated epithelium^27,28^, but may be less relevant to interactions of intra-tumor bacteria. On the other hand, 3D tumor spheroids, which were used in this study, serve as a representative *in vitro* model of the TME^29–31^. However, BSCCs are less likely to be useful in generally studying the vast microbiota interactions of the native intestinal lumen environment.

To characterize this model, we first demonstrated that *F*. *nucleatum* 23726 and 25586 consistently remained viable in 40,000 cell spheroids through 48 h. This *F*. *nucleatum* viability is likely in part due to the hypoxic niche that is characteristic of large spheroids^35^. Upon observing significant biomass increase (sometimes visible to the naked eye) concurrent with tumor spheroid fragility, we characterized the kinetics of *F*. *nucleatum* growth and tumor spheroid cytotoxicity. Using fluorescence confocal microscopy, we observed biofilm-like aggregate formation and altered tumor spheroid morphology as the BSCC progressed, which revealed that at 24 and 36 h viable *F*. *nucleatum* are primarily seen in extracellular aggregates in the tumor spheroid microenvironment, whereas HK *F*. *nucleatum* are internalized into the cytoplasm of the HT29 cells. This suggests that proliferating *F*. *nucleatum* either escape the cancer cells after internalization, avoid endocytosis by forming and residing in biofilm, or perhaps a combination of both. Indeed, evidence of bacterial biofilm in colon cancer has been previously reported^10,42,46,47^, further supporting that this is an important characteristic of bacteria in this niche. Future identification of biofilm components of *F*. *nucleatum* (or other bacteria) that are present in the TME could lead to new discoveries of how these bacteria interact with the tumor.

To build upon these qualitative findings from fluorescence microscopy, we took approaches toward molecular quantification of the interactions between *F*. *nucleatum* and the tumor spheroids, via high-throughput qPCR (and later on using high resolution metabolomics). We measured cancer-related gene expression in response to proliferating *F*. *nucleatum* 23726 and 25586 before substantial cytotoxicity was observed. The transcriptional response of HT29 cells to both viable *F*. *nucleatum* strains was very similar; perhaps unsurprisingly, as these strains are genetically quite similar^45^. Colorectal cancer metastasis signaling, IL-8 signaling, and molecular mechanisms of cancer were highly enriched in response to both viable strains. The latter (molecular mechanisms of cancer) may be unsurprising as the gene panel was based on cancer related pathways, but it is worth noting that this was not enriched in BSCCs with HK *F*. *nucleatum*. Increased IL-8 gene expression has been observed in patients with high *Fusobacterium* abundance^16^, and increased IL-8 protein levels were measured in BSCCs with viable *F*. *nucleatum* in this study. Interestingly, *F*. *nucleatum* has been associated with metastasis in multiple reports^6,18,19,44^ and even cultured from metastatic lesions^10^. Several of the most highly enriched canonical pathways from HK 23726-treated BSCCs (e.g. PTEN, STAT3, viral entry via endocytosis) were also significantly enriched (*p* < 0.001, Fisher’s exact test) in BSCCs with viable strains, and *F*. *nucleatum* has been previously connected to these pathways. *F*. *nucleatum* has been reported to down-regulate PTEN expression in epithelial cancer cells and induce STAT3 expression in macrophages, both via TLR4-dependent mechanisms^14,48^. *F*. *nucleatum* has also been shown to internalize via clathrin-mediated endocytosis upon binding E-cadherin with its FadA adhesin^13^. This suggests that the BSCC with viable *F*. *nucleatum* captures some of the signaling pathways that occur with HK *F*. *nucleatum*, likely a result of bacterial surface components. A limitation of our transcriptomic analysis was that the cancer-related gene panel only provides a focused subset of the transcriptome and it is likely that unbiased transcriptomic analyses (i.e. RNA-sequencing) would reveal additional pathways affected. While we are employing this co-culture model to better understand the effects of viable *F*. *nucleatum*, we must also point out that the bacterial density as a result of proliferation can also be a confounder in the experiment. At 24 h, both strains are entering logarithmic growth phase (Fig. 2A) and therefore may be at a different density than the HK *F*. *nucleatum*.

We also characterized the metabolite content of BSCCs at various timepoints using high resolution metabolomics to gain an understanding of how viable *F*. *nucleatum* may influence metabolite levels and availability in the TME. From these experiments, we made two observations in this BSCC model that parallel what has been previously reported in non-coculture systems. First, we saw a depletion of six amino acids: histidine, glutamine, serine, methionine, threonine, and tryptophan. The former five of these have been reported as energy substrates in *F*. *nucleatum*^40,41^. The latter, tryptophan, has been shown to induce biofilm formation in *F*. *nucleatum* 25586 (which was seen in the BSCC), potentially through the production of the extracellular signaling molecule indole^49^. We also observed an increase in the polyamines N^1^-acetylspermidine and N^1^,N^12^-diacetylspermine. Polyamines are important mediators in colon carcinogenesis, and polyamine metabolism has been a target of preclinical and clinical chemoprevention studies going back decades^50,51^. Interestingly, Johnson *et al*. observed increased levels of polyamines in patient-derived colorectal tumors that were biofilm-positive (in comparison to biofilm-negative tumors), and hypothesized that these metabolites were being contributed by the biofilm community^42^. While our observations in the BSCC model did not distinguish which species was utilizing or providing these metabolites (e.g. host vs. microbe), it establishes a platform for addressing these questions.

In order to more closely mimic the TME, tumor spheroid models have been developed to co-culture cancer cells with endothelial cells^52,53^, fibroblasts^53,54^, stellate cells^55^, or various immune cells^56–60^; and three-way co-cultures are emerging^61,62^. While evidence for the influence of intratumor bacteria on cancer progression builds, this BSCC with colorectal cancer-associated intratumor bacteria adds a novel model to study this disease. While this current BSCC model only incorporates bacteria and cancer cells, these different models are continuously being integrated offering potential for future characterization of how intratumor bacteria affect other cell types in the TME beyond cancer cells. Although the CRC field has the most established evidence of a tumor microbiome, other tumor microbiomes are emerging such as in pancreatic cancers^63,64^, indicating this tumor-microbe relationship is likely not limited to CRC. Lastly, as *F*. *nucleatum* is not the only bacterium reported in the CRC TME, this model offers potential for studying other bacterial species and consortia in a reductionist system for more complete understanding of interspecies and interkingdom ecologies in the context of colorectal and other cancers.

## Methods

### Cell culture, tumor spheroid formation, bacterial culture, and bacteria/spheroid co-culture (BSCC)

The human epithelial colon cancer cell lines HT-29 and HCT116 were purchased from American Type Culture Collection (ATCC, Manassas, VA, USA). HT-29 eGFP was purchased from Genecopoeia (Cat No. SL106, Rockville, MD, USA). All cell lines were maintained in McCoy’s 5 A Medium (ATCC, 30–2007) with 10% fetal bovine serum (FBS, Gibco 10082-139), and antibiotics (penicillin (100 units/ml) and streptomycin (100 μg/ml), Gibco 15140-122) in 5% CO_2_ at 37 °C.

Tumor spheroids were formed by resuspending cells in fresh medium without antibiotics and aliquoting 200 μl per well of a 96-well ultra-low attachment spheroid microplate (Corning, Cat No. 4520). Tumor spheroids were incubated for 48 h at 37 °C, 5% CO_2_, before inoculating with bacteria.

Bacterial strains used in this study are listed in Supplementary Table 1. All *Fusobacterium* strains were streaked from frozen stock on brain-heart infusion (BHI) agar (AS-6226, Anaerobe Systems, Morgan Hill, CA, USA) in an anerobic chamber (Coy Laboratory Products, Grass Lake, MI, USA) and incubated at 37 °C in the chamber. *Faecalibacterium prausnitzii* was streaked out onto yeast casitone fatty acids agar with carbohydrates (YCFAC, AS-675, Anaerobe Systems) in an anaerobic chamber and incubated at 37 °C. Liquid cultures of all bacterial strains were made by inoculating a single colony into 5 ml BHI broth (AS-872, Anaerobe Systems). Liquid cultures were incubated anaerobically for 48 h at 37 °C before use, yielding bacterial densities of approximately 18 × 10^6^ CFUs/ml for ATCC 23726 and 4 × 10^6^ CFUs/ml for ATCC 25586. To initiate BSCCs, liquid cultures were pelleted down, supernatant was discarded, pellets were resuspended in the same volume of BHI broth, and 4 μL of suspension were transferred to each well of the tumor spheroid microplate (MOIs of approximately 1.8:1 and 0.4:1 for ATCC 23726 and ATCC 25586, respectively). BSCCs were then incubated at 37 °C, 5% CO_2_ (standard humidified incubator) from this point forward.

### Live cell imaging

BSCCs were imaged using two separate live cell imaging platforms: Incucyte S3 Live-cell Analysis System and Biotek Cytation 5 Cell Imaging Multi-mode Reader, using standard brightfield, phase contrast, and fluorescence protocols.

### Cell lysis assay

The CellTox™ Green (Promega, G8741) cytoxicity assay was used according to manufacturer’s protocol with slight modification. CellTox™ Green Dye (1000×) was diluted 10-fold in fresh media (without antibiotics). BSCCs were initiated as described above. Vehicle control was 4 μL of sterile BHI added to tumor spheroid. The lysis solution accompanying the kit was added at 4 μL per well. At the time of adding *F*. *nucleatum*/controls, 20 μL of CellTox Green Dye/cell media solution was added to each BSCC. Plates were placed in Incucyte S3 Live-cell Analysis System and whole-well imaged for both phase contrast and green fluorescence (acquisition time 300 ms) every 6 h. Green fluorescence signal in the images were analyzed using Incucyte software. Fixed threshold segmentation was applied (600 GCU threshold) and data is displayed as total green object integrated intensity (GCU × μm^2^/well).

### 16S quantification of bacterial growth

DNA was isolated from each BSCC using the Qiagen QIAamp PowerFecal DNA Kit (12830-50) following manufacturer’s instructions. Bacterial DNA abundance was assessed by qPCR amplification of the V1-V2 region of the 16S rRNA gene using the Taqman Fast Advanced qPCR master mix (Thermo Fisher 4444963) as previously described^65^. qPCR reactions were carried out in triplicate (20 μL each). Degenerate bacterial 16S rDNA-specific primers and probe (Sigma) with the following sequences were used: forward primer, 5′AGAGTTTGATCCTGGCTCAG3′; reverse primer, 5′-CTGCTGCCTYCCGTA-3′; probe: 5′-/56-FAM/TAA + CA + CATG + CA + AGT + CGA/3BHQ_1/3′, “+” indicates the position of an LNA base. A standard curve was prepared using a near full length amplicon of *Escherichia coli* 16S rRNA gene inserted into a Topo vector for normalization. Thermocycling was carried out using a QuantStudio 12 K Flex (Applied Biosystems) as follows: initiation at 95 °C for 5 min followed by 40 cycles of 94 °C × 30 s, 50 °C × 30 s, and 72 °C × 30 s.

### IL-8 quantification

After 24 h of co-culture, supernatants were collected and pooled for each BSCC. Each pooled supernatant was filtered using a 0.2 μm spin filter (Costar) in microcentrifuge tubes. Filtered supernatants were used to measure IL-8 concentrations using a Simple Plex Cartridge kit from Protein Simple with their Ella Simple Plex instrument. Briefly, 50 μL of the supernatants was added to the sample wells in addition to the high and low controls included in the kit. The Simple Plex Runner and Explorer softwares were used to setup the assay and analyze the results, respectively.

### Confocal laser scanning microscopy

Each BSCC condition was pooled in a microcentrifuge tube, allowed to settle to the bottom, and media was removed. BSCCs were fixed with 4% paraformaldehyde (v/v) in PBS and permeabilized using a 1% (v/v) Triton X (Invitrogen 28314) solution in tris-buffered saline (TBS) followed by a blocking step using a solution of 5% (v/v) BSA in TBS. BSCCs were stained using Hoechst (Life Technologies) to stain for cell nuclei. For tubulin visualization, a mouse anti-tubulin antibody (Sigma T6199) with an Alexa Fluor 488 goat anti-mouse secondary antibody (Invitrogen A11029) was used. *F*. *nucleatum* 25586 was stained with a rabbit anti-*F*.*nucleatum* 25586 polyclonal antibody mix (Diatheva ANT0084) which was detected with an Alexa Fluor 647-conjugated donkey anti-rabbit IgG secondary antibody (Invitrogen A31573). Labeling of BSCCs with *F*. *nucleatum* 23726 was also attempted, however, the polyclonal anti-*F*. *nucleatum* antibodies did not label this strain well in our hands. CLSM was performed with a Zeiss LSM 800 Axio Observer.Z1/7 using the Zen 2.5 software with the following settings: excitation at 353 nm, emission at 410-470 nm for Hoechst; excitation at 488 nm, emission at 485–565 nm for tubulin; and excitation at 631 nm, emission at 644–700 nm to detect *F*. *nucleatum*.

### High-throughput RT-qPCR

BSCCs were initiated as described above with the following treatments: BHI (vehicle), *F*. *nucleatum* 23726, *F*. *nucleatum* 25586, and HK *F*. *nucleatum* 23726. After 24 h of co-culture, groups of four BSCCs were pooled in triplicate, except for HK *F*. *nucleatum* 23726, which had duplicate pools. RNA isolation was carried out for each sample set following the RNeasy Mini Kit protocol (Qiagen 74104). DNase (Qiagen 79254) treatment was performed on the purification column following manufacturer’s instructions. Isolated RNA was used as template for transcriptional profiling of genes involved in key pathways of cancer using the TaqMan OpenArray Human Cancer Panel (Applied Biosystems 4475391), which consists of 648 TaqMan qPCR assays arranged on an OpenArray plate. The TaqMan® OpenArray® Pathway Panels low sample input protocol (10–50 ng/μL) was followed. Gene-specific reverse transcription was performed using SuperScript™ VILO™ cDNA Synthesis Kit (Thermo Fisher 11754050) and TaqMan PreAmp primer pools A and B (Thermo Fisher 4485255). Pre-amplification of cDNA was performed by combining cDNA, primer pools, and TaqMan PreAmp mastermix (Thermo Fisher 4391128), and subsequent thermal cycling. Pool A and Pool B for each sample was combined 1:1 and then diluted 1:20 into nuclease-free water. Mixed pre-amplification products were mixed 1:1 with 2x TaqMan OpenArray real-time mastermix (Thermo Fisher 4462164) and loaded onto the OpenArray plate using the OpenArray AccuFill System (Thermo Fisher 4457243). Loaded OpenArray plates were placed in QuantStudio™ 12 K Flex Real-time PCR system for qPCR thermal cycling. Data was analyzed in ExpressionSuite software, where dCt and relative quantification (compared against vehicle control) were calculated using B2M, UBC, and YWHAZ as endogenous controls. Unpaired t-tests for each treatment vs. control were calculated using GraphPad Prism. Heatmaps were made using Heatmapper^66^ and Venn diagrams were made using BioVenn^67^.

### Pathway Analysis

Differentially expressed genes (*p* < 0.05, unpaired T-test) for each treatment group were uploaded into Qiagen Ingenuity Pathway Analysis (IPA) software for further analysis and interpretation. Default analysis settings were used except for species (“human” was used) and tissues and cell lines (“colon cancer cell lines” was used). Disease/function networks shown are *p* < 0.0001 (Fisher’s exact test) except for BSCC with *F*. *nucleatum* 25586 where networks shown are *p* < 0.0001 and have calculated Z-scores (33 total disease/functions were *p* < 0.0001 for this treatment).

### Metabolomic analysis

Samples were analyzed by targeted and untargeted LC/MS analysis. For targeted analysis, an Agilent 6470 Triple Quadrupole (QQQ) mass spectrometer (Agilent Technologies, Santa Clara, CA), in negative ionization mode, coupled to an Agilent 1290 Infinity II HPLC with quaternary pump was used. Metabolites were separated using an Agilent ZORBAX RRHD Extend-C18 (2.1 × 150 mm, 1.8 µm) column with the following mobile phases: (A) H_2_O:methanol (97:3) with 15 mM glacial acetic acid and 10 mM tributylamine; (B) methanol with 15 mM glacial acetic acid and 10 mM tributylamine; (D) acetonitrile. Multiple Reaction Monitoring (MRM) transitions for the central carbon metabolites were from the Agilent Metabolomics MRM Database and Method. For untargeted analysis, an Agilent 6545 Quadrupole Time-of-Flight (QTOF) mass spectrometer coupled to an Agilent 1290 Infinity II HPLC with binary pump was used. Metabolites were separated using an Agilent InfinityLab Poroshell 120 HILIC-Z (2.1 × 100 mm, 2.7 μm) column using the following mobile phases: (A) 200 mM ammonium formate (pH 3): H_2_O (10:90); (B) 90:10, ACN:H_2_O with 200 mM ammonium formate, pH 3 with 5 µM medronic acid. QTOF MS data was acquired by ESI positive mode with a mass range of 62–1000 Da. For targeted metabolomics, data was analyzed using Agilent MassHunter Quantitative Analysis B.08 software. For untargeted metabolomics, data was analyzed using Agilent MassHunter Profinder software and Mass Profiler Professional version 14.9.1. Additional method information is described in Supplementary Tables 3 and 4.

For each sample, four BSCCs in media were pipetted onto a PTFE membrane filter (0.45 μm pore size, 47 mm diameter; EMD Millipore JHWP04700) under vacuum using glass base apparatus (Fisher XX1014702). Media was allowed to pass through filter, and BSCCs remained on top of filter. Filter paper containing cells were placed in a 60 mm diameter petri dish (cells facing down) containing 1.5 mL ice cold extraction solvent (acetonitrile: methanol: water; 40:40:20) and allowed to extract for 20 minutes at −20 °C (placed on top of ice/dry ice). After 20 minutes, extraction solvent was transferred to a clean 1.5 mL microcentrifuge tube, and tubes were centrifuged for 10 minutes at 4 °C at 15,000 × g. Supernatant (1000 µL) was transferred to a clean 1.5 mL microcentrifuge tube, dried under nitrogen, and re-dissolved in 60 µL water:methanol (80:20) for LC/MS analysis.

## Supplementary information

Supplementary Information.

## References

[1] Siegel RL, Miller KD, Jemal A (2017). Cancer statistics, 2017. CA. Cancer J. Clin..

[2] Aran V, Victorino AP, Thuler LC, Ferreira CG (2016). Colorectal Cancer: Epidemiology, Disease Mechanisms and Interventions to Reduce Onset and Mortality. Clin. Colorectal Cancer.

[3] Sears CL, Garrett WS (2014). Microbes, microbiota, and colon cancer. Cell Host Microbe.

[4] Louis P, Hold GL, Flint HJ (2014). The gut microbiota, bacterial metabolites and colorectal cancer. Nat. Rev. Microbiol..

[5] Zackular JP, Baxter NT, Chen GY, Schloss PD (2015). Manipulation of the Gut Microbiota Reveals Role in Colon Tumorigenesis. mSphere.

[6] Castellarin M (2012). Fusobacterium nucleatum infection is prevalent in human colorectal carcinoma. Genome Res..

[7] Kostic AD (2012). Genomic analysis identifies association of Fusobacterium with colorectal carcinoma. Genome Res..

[8] Flanagan L (2014). Fusobacterium nucleatum associates with stages of colorectal neoplasia development, colorectal cancer and disease outcome. Eur. J. Clin. Microbiol. Infect. Dis..

[9] Ito M (2015). Association of Fusobacterium nucleatum with clinical and molecular features in colorectal serrated pathway. Int. J. Cancer.

[10] Bullman S (2017). Analysis of Fusobacterium persistence and antibiotic response in colorectal cancer. Science.

[11] Brennan CA, Garrett WS (2019). Fusobacterium nucleatum — symbiont, opportunist and oncobacterium. Nat. Rev. Microbiol..

[12] Flynn KJ, Baxter NT, Schloss PD (2016). Metabolic and Community Synergy of Oral Bacteria in Colorectal Cancer. mSphere.

[13] Rubinstein MR (2013). Fusobacterium nucleatum Promotes Colorectal Carcinogenesis by Modulating E-Cadherin/β-Catenin Signaling via its FadA Adhesin. Cell Host Microbe.

[14] Yang Y (2017). Fusobacterium nucleatum Increases Proliferation of Colorectal Cancer Cells and Tumor Development in Mice by Activating Toll-Like Receptor 4 Signaling to Nuclear Factor−κB, and Up-regulating Expression of MicroRNA-21. Gastroenterology.

[15] Gur C (2015). Binding of the Fap2 protein of Fusobacterium nucleatum to human inhibitory receptor TIGIT protects tumors from immune cell attack. Immunity.

[16] Kostic AD (2013). Fusobacterium nucleatum Potentiates Intestinal Tumorigenesis and Modulates the Tumor-Immune Microenvironment. Cell Host Microbe.

[17] Yu TC (2017). Fusobacterium nucleatum Promotes Chemoresistance to Colorectal Cancer by Modulating Autophagy. Cell.

[18] Yan X, Liu L, Li H, Qin H, Sun Z (2017). Clinical significance of Fusobacterium nucleatum, epithelial–mesenchymal transition, and cancer stem cell markers in stage III/IV colorectal cancer patients. Onco. Targets. Ther..

[19] Li YY (2016). Association of Fusobacterium nucleatum infection with colorectal cancer in Chinese patients. World J. Gastroenterol..

[20] Fritz JV, Desai MS, Shah P, Schneider JG, Wilmes P (2013). From meta-omics to causality: Experimental models for human microbiome research. Microbiome.

[21] von Martels JZH (2017). The role of gut microbiota in health and disease: *In vitro* modeling of host-microbe interactions at the aerobe-anaerobe interphase of the human gut. Anaerobe.

[22] Sadabad MS (2015). A simple coculture system shows mutualism between anaerobic faecalibacteria and epithelial Caco-2 cells. Sci. Rep..

[23] Shah, P. *et al*. A microfluidics-based *in vitro* model of the gastrointestinal human-microbe interface. *Nat*. *Commun*. **7** (2016). 10.1038/ncomms11535 PMC4865890 27168102

[24] Eain MMG (2017). Engineering Solutions for Representative Models of the Gastrointestinal Human-Microbe. Interface. Engineering.

[25] Ulluwishewa D (2015). Live Faecalibacterium prausnitzii in an apical anaerobic model of the intestinal epithelial barrier. Cell. Microbiol..

[26] Jalili-Firoozinezhad S (2019). A complex human gut microbiome cultured in an anaerobic intestine-on-a-chip. Nat. Biomed. Eng..

[27] Leslie JL (2015). Persistence and toxin production by Clostridium difficile within human intestinal organoids result in disruption of epithelial paracellular barrier function. Infect. Immun..

[28] Williamson IA (2018). A High-Throughput Organoid Microinjection Platform to Study Gastrointestinal Microbiota and Luminal Physiology. Cell. Mol. Gastroenterol. Hepatol..

[29] Costa EC (2016). 3D tumor spheroids: an overview on the tools and techniques used for their analysis. Biotechnol. Adv..

[30] Hirschhaeuser F (2010). Multicellular tumor spheroids: an underestimated tool is catching up again. J. Biotechnol..

[31] Zanoni M (2016). 3D tumor spheroid models for *in vitro* therapeutic screening: a systematic approach to enhance the biological relevance of data obtained. Sci. Rep..

[32] Osswald A (2015). Three-dimensional tumor spheroids for *in vitro* analysis of bacteria as gene delivery vectors in tumor therapy. Microb. Cell Fact..

[33] Harimoto, T. *et al*. Rapid screening of engineered microbial therapies in a 3D multicellular model. *Proc*. *Natl*. *Acad*. *Sci*. **116** (2019). 10.1073/pnas.1820824116 PMC6500119 30996123

[34] Nakatsu G (2015). Gut mucosal microbiome across stages of colorectal carcinogenesis. Nat. Commun..

[35] Sutherland RM (1986). Oxygen and Differentiation in Multicellular Spheroids of Human Colon Carcinoma. Cancer Res..

[36] McCoy, A. N. *et al*. Fusobacterium Is Associated with Colorectal Adenomas. *PLoS One* **8** (2013). 10.1371/journal.pone.0053653 PMC3546075 23335968

[37] Wong, S. H. *et al*. Quantitation of faecal Fusobacterium improves faecal immunochemical test in detecting advanced colorectal neoplasia. *Gut* 1441–1448, 10.1136/gutjnl-2016-312766 (2017). 10.1136/gutjnl-2016-312766 PMC5530471 27797940

[38] Mima K (2015). Fusobacterium nucleatum and T cells in Colorectal Carcinoma. JAMA Oncol..

[39] Mima, K. *et al*. Fusobacterium nucleatum in colorectal carcinoma tissue and patient prognosis. *Gut* 1973–1980, 10.1136/gutjnl-2015-310101 (2016). 10.1136/gutjnl-2015-310101 PMC4769120 26311717

[40] Kapatral V (2005). Genome sequence and analysis of the oral bacterium Fusobacterium nucleatum Strain ATCC 25586. J. Bacteriol..

[41] Bolstad AI, Jensen HB, Bakken V (1996). Taxonomy, Biology, and Periodontal Aspects of Fusobacterium nucleatum. Clin. Microbiol. Rev..

[42] Johnson CH (2015). Metabolism links bacterial biofilms and colon carcinogenesis. Cell Metab..

[43] Hold GL (2016). Gastrointestinal Microbiota and Colon Cancer. Dig. Dis..

[44] Abed J (2016). Fap2 Mediates Fusobacterium nucleatum Colorectal Adenocarcinoma Enrichment by Binding to Tumor-Expressed Gal-GalNAc. Cell Host Microbe.

[45] McGuire AM (2014). Evolution of Invasion in a Diverse Set of Fusobacterium Species. MBio.

[46] Dejea CM (2014). Microbiota organization is a distinct feature of proximal colorectal cancers. Proc. Natl. Acad. Sci..

[47] Dejea CM (2018). Patients with familial adenomatous polyposis harbor colonic biofilms containing tumorigenic bacteria. Science.

[48] Chen T (2018). Fusobacterium nucleatum promotes M2 polarization of macrophages in the microenvironment of colorectal tumours via a TLR4-dependent mechanism. Cancer Immunol. Immunother..

[49] Sasaki-Imamura T, Yano A, Yoshida Y (2010). Production of indole from L-tryptophan and effects of these compounds on biofilm formation by fusobacterium nucleatum ATCC 25586. Appl. Environ. Microbiol..

[50] Gerner EW, Bruckheimer E, Cohen A (2018). Cancer pharmacoprevention: Targeting polyamine metabolism to manage risk factors for colon cancer. J. Biol. Chem..

[51] Gerner, E. W. & Meyskens Jr., F. L. Polyamines and cancer: old molecules, new understanding. *Nat*. *Rev*. *Cancer* **4** (2004). 10.1038/nrc1454 15510159

[52] Upreti M (2011). Tumor-Endothelial Cell Spheroids: New Aspects to Enhance Radiation. Transl. Oncol..

[53] Cattin S, Ramont L, Rüegg C (2018). Characterization and *In Vivo* Validation of a Three-Dimensional Multi-Cellular Culture Model to Study Heterotypic Interactions in Colorectal Cancer Cell Growth, Invasion and Metastasis. Front. Bioeng. Biotechnol..

[54] Jeong S, Lee J, Shin Y, Chung S, Kuh H (2016). Co-Culture of Tumor Spheroids and Fibroblasts in a Collagen Matrix-Incorporated Microfluidic Chip Mimics Reciprocal Activation in Solid Tumor Microenvironment. PLoS One.

[55] Chen Y (2019). Microfluidic co-culture of liver tumor spheroids with stellate cells for the investigation of drug resistance and intercellular interactions. Analyst.

[56] Long, L., Yin, M. & Min, W. 3D Co-culture System of Tumor-associated Macrophages and Ovarian Cancer Cells. *Bio-protocol* **8** (2018). 10.21769/BioProtoc.2815 PMC5951403 29770354

[57] Sherman H, Gitschier HJ, Rossi AE (2018). A Novel three-dimensional Immune oncology Model for high-throughput testing of tumoricidal Activity. Front. Immunol..

[58] Courau T (2019). Cocultures of human colorectal tumor spheroids with immune cells reveal the therapeutic potential of MICA/B and NKG2A targeting for cancer treatment. *J*. *Immunother*. Cancer.

[59] Trumpi K (2018). Macrophages induce “budding” in aggressive human colon cancer subtypes by protease-mediated disruption of tight junctions. Oncotarget.

[60] Varesano, S., Zocchi, M. R. & Poggi, A. Zoledronate Triggers Vδ2 T Cells to Destroy and Kill Spheroids of Colon Carcinoma: Quantitative Image Analysis of Three-Dimensional Cultures. *Front*. *Immunol*. **9** (2018). 10.3389/fimmu.2018.00998 PMC5951939 29867975

[61] Lazzari G (2018). Multicellular spheroid based on a triple co-culture: A novel 3D model to mimic pancreatic tumor complexity. Acta Biomater..

[62] Susanti S (2018). PO-304 Three-dimensional co-culture of colorectal cancer spheroid with cancer-associated fibroblast as a model to study immune cell modulation. ESMO Open.

[63] Geller LT (2017). Potential role of intratumor bacteria in mediating tumor resistance to the chemotherapeutic drug gemcitabine. Science.

[64] Riquelme E (2019). Tumor Microbiome Diversity and Composition Influence Pancreatic Cancer Outcomes. Cell.

[65] Liang X, Bushman FD, Fitzgerald GA (2015). Rhythmicity of the intestinal microbiota is regulated by gender and the host circadian clock. Proc. Natl. Acad. Sci..

[66] Babicki S (2016). Heatmapper: web-enabled heat mapping for all. Nucleic Acids Res..

[67] Hulsen T, de Vlieg J, Alkema W (2008). BioVenn – a web application for the comparison and visualization of biological lists using area-proportional Venn diagrams. BMC Genomics.

