# Supplementary material for: Colorectal cancer-associated anaerobic bacteria proliferate in tumor spheroids and alter the microenvironment
Source: Sci Rep. 2020 Mar 24;10:5321. doi: 10.1038/s41598-020-62139-z (PMC7093526; doi:10.1038/s41598-020-62139-z)
Supplement: Supplementary file 1 — Supplementary Information. [file 41598_2020_62139_MOESM1_ESM.doc]

Supplementary Information

**Colorectal cancer-associated anaerobic bacteria proliferate in tumor spheroids and alter the microenvironment**

Stephen H. Kasper^1,2^,*, Carolina Morell-Perez^1,2^, Thomas P. Wyche^1^, Theodore R. Sana^1^, Linda A. Lieberman^1^, Erik C. Hett^1^,*

^1^ Exploratory Science Center, Merck & Co., Inc., Cambridge, Massachusetts, USA

^2^ These authors contributed equally to this work

* Corresponding authors (stephen.kasper@merck.com, erik.hett@merck.com)

**SUPPLEMENTARY FIGURES**

| **Supplementary Table 1**. Bacterial strains used in this study | | | | |
| --- | --- | --- | --- | --- |
| Strain |  | Description/isolation |  | Source or reference |
| *Faecalibacterium prausnitzii* |  |  |  |  |
| 27768 (VPI C13-51) |  | Human feces |  | ATCC |
| *Fusobacterium nucleatum* |  |  |  |  |
| EAVG_005 (13_3C) |  | ascending colon |  | [45], * |
| EAVG_009 (3_2_4) |  | ileum |  | [45], * |
| EAVO_002 (1_A_7) |  | mouth, inflamed biopsy tissue (Crohn's) |  | [45], * |
| EAVG_012 (4_1_13) |  | ascending colon, healthy biopsy tissue |  | [45], * |
| EAVO_006 (2_A_13) |  | mouth |  | [45], * |
| EAVO_005 (1_A_13) |  | mouth |  | [45], * |
| EAVG_029 (4_1_31B) |  | ascending colon |  | [45], * |
| EAVG_015 (2_1_31) |  | ascending colon, inflamed biopsy tissue (Crohn's) |  | [45], * |
| EAVG_018 (3_1_36A2) |  | ascending colon |  | [45], * |
| EAVO_009 (1_A_36) |  | mouth, inflamed biopsy tissue (Crohn's) |  | [45], * |
| EAVO_010 (2_A_37) |  | mouth |  | [45], * |
| EAVG_021 (3_1_37BFAA) |  | terminal ileum |  | [45], * |
| EAVG_023 (3_2_44B) |  | descending colon |  | [45], * |
| EAVG_025 (3_1_48A) |  | distal ileum |  | [45], * |
| EAVG_027 (2_1_50A) |  | distal colon |  | [45], * |
| Subsp. *nucleatum* 23726 (VPI 4351 [1210]) |  |  |  | ATCC |
| Subsp. *nucleatum* 25586 (VPI 4355 [1612A]) |  | cervico-facial lesion |  | ATCC |
| Subsp. *nucleatum* CC2_3FMU1 |  | colon, colorectal cancer patient |  | * |
| Subsp. *animalis* D11 |  | intestine, healthy biopsy tissue (Crohn's) |  | * |
| Subsp. *animalis* EAVG_001 (4_8) |  | sigmoid colon, healthy biopsy tissue |  | [45], * |
| Subsp. animalis EAVG_002 (7_1) |  | sigmoid colon |  | [45], * |
| Subsp. *animalis* EAVG_003 (11_3_2) |  | non-specific colon |  | [45], * |
| Subsp. *animalis* CC2_6JVN3 |  | colon, colorectal cancer patient |  | * |
| Subsp. *animalis* CC7_3JVN3C1 |  | colon, colorectal cancer patient |  | * |
| *Fusobacterium periodonticum* |  |  |  |  |
| EAVG_028 (27_1) |  | terminal ileum |  | [45], * |
| EAVG_011 (3_1_7B) |  | sigmoid colon |  | [45], * |
| *Fusobacterium necrophorum* |  |  |  |  |
| EAVG_019 (1_1_36S) |  | terminal ileum, inflamed biopsy tissue (Crohn's) |  | [45], * |
| EAVG_026 (D12 or 3_1_49) |  | cecum, inflamed biopsy tissue (UC patient) |  | [45], * |
| *Fusobacterium gonidiaformans* |  |  |  |  |
| EAVG_010 (3_1_5R) |  | intestine, inflamed biopsy tissue (UC patient) |  | [45], * |
| *Fusobacterium ulcerans* |  |  |  |  |
| EAVG_004 (12_1B) |  | colon, inflamed biopsy tissue (Crohn's) |  | [45], * |

* indicates strains that were provided by Professor Emma Allen-Vercoe at University of Guelph

**Supplementary Figure 1**. (**TOP**) Recovery of viable *F. nucleatum* and lack of recovery of viable *F. prausnitzii*. Viable bacteria recovered at 24 h and 48 h are indicated by dark circles with bold outline. (**BOTTOM**) Phase contrast images of the BSCC model at 0 h, 24 h and 48 h post inoculation with *F. nucleatum* 23726, 25586, *F. prausnitzii*, or vehicle. White arrows indicate the enlarged biomass seen at 48 h in 40,000 cell spheroids inoculated with viable *F. nucleatum*. For scale, tumor spheroids are in a 6.35 mm diameter well (outer most curve).


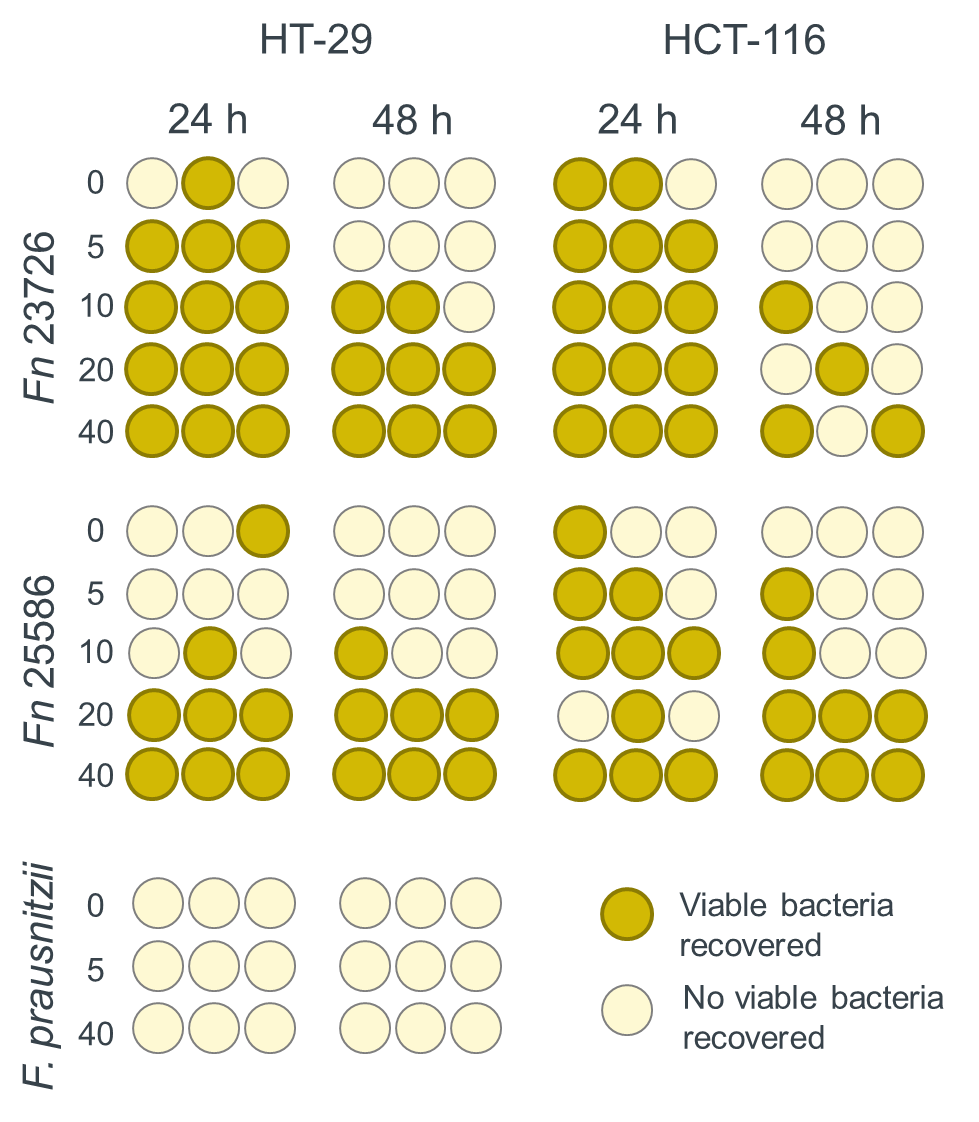


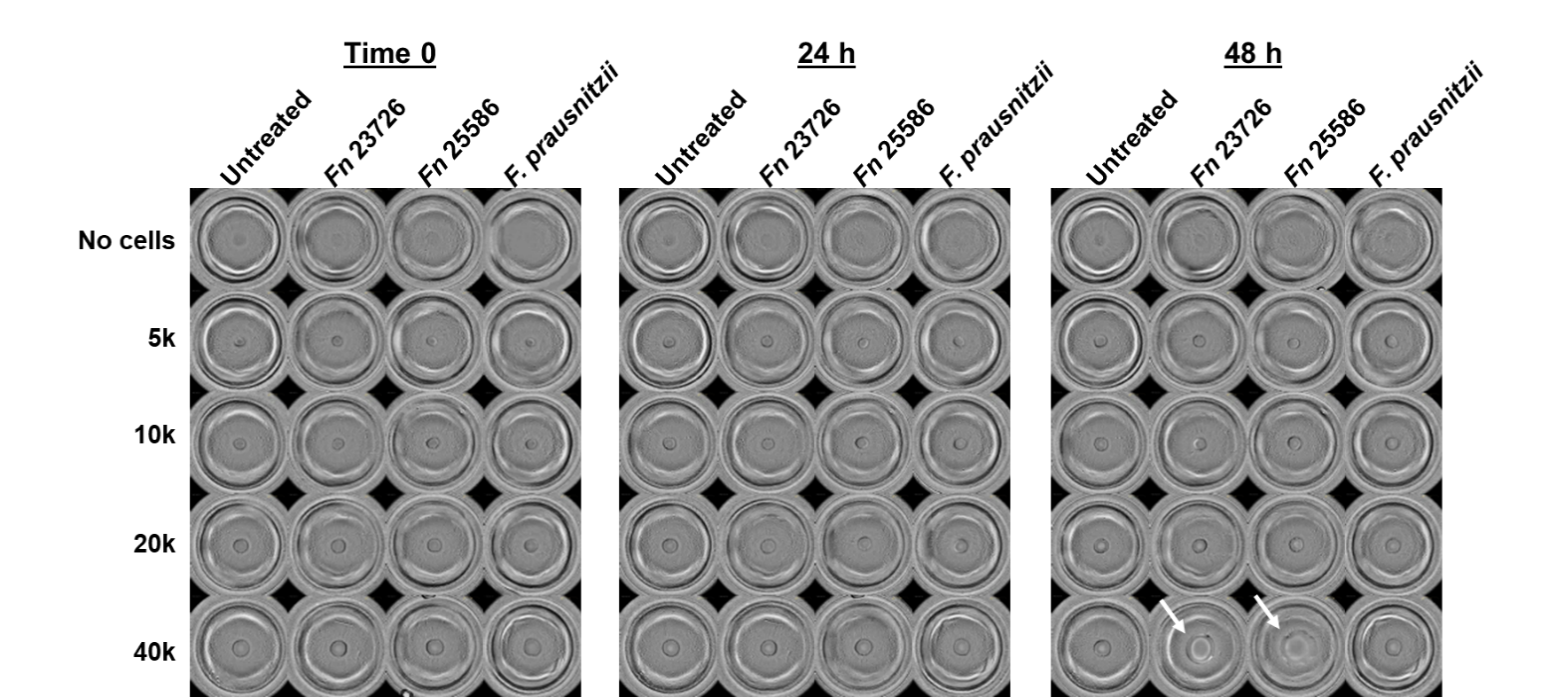


**Supplementary Figure 2**. Growth of *F. nucleatum* 23726 (blue) and *F. nucleatum* 25586 (red) in human cell media in aerobic conditions (no spheroid, left), human cell media in anaerobic conditions (center), and bacterial media in anaerobic conditions (right).


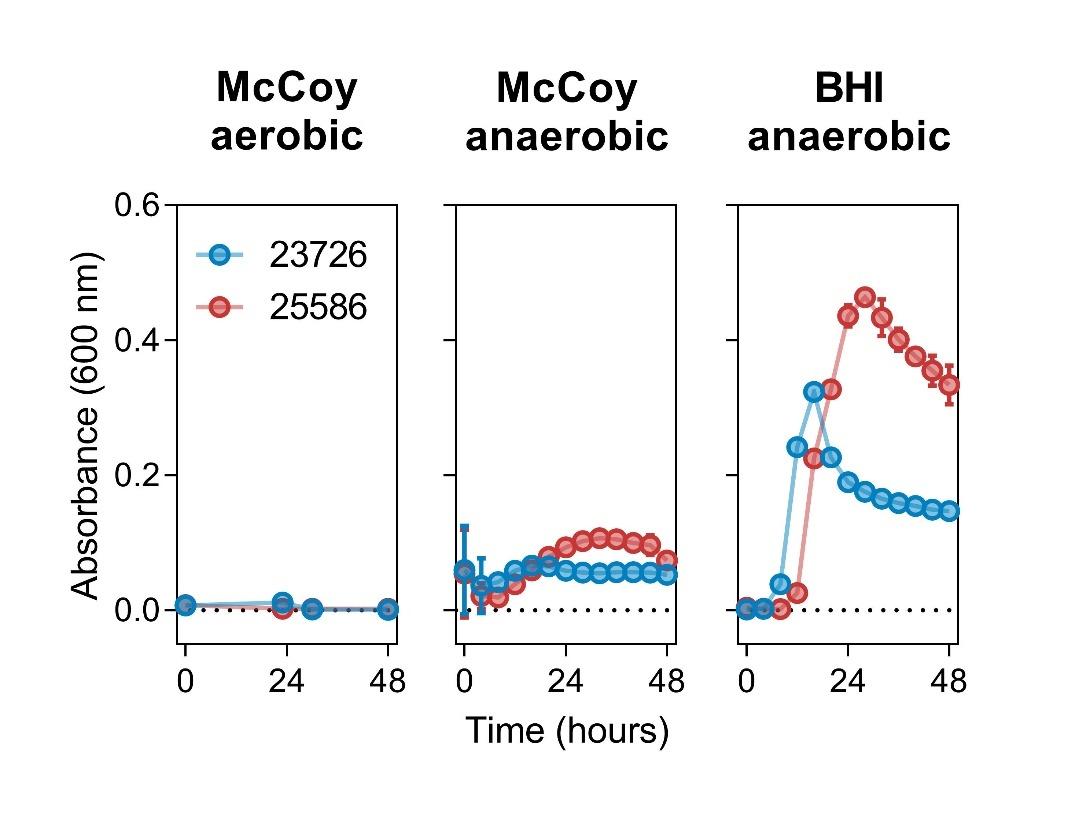


**Supplementary Figure 3.** Venn diagram representing the number and overlap of differentially expressed genes (DEGs) in BSCCs with *F. nucleatum* 23726, 25586, or HK *F. nucleatum* 23726. Complete list of DEGs can be found in Supplementary Table 2.


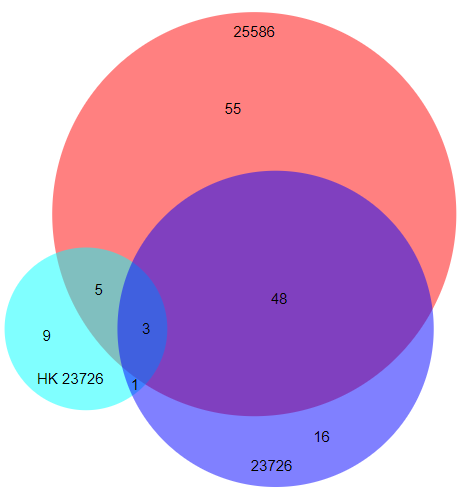


**Supplementary Table 2**. Table of differentially expressed genes (*p* < 0.05, unpaired T test). Fold changes of two or greater are shown in bold.

| **Symbol** | **Entrez Gene Name** | **HK 23726** | | **23726** | | **25586** | |
| --- | --- | --- | --- | --- | --- | --- | --- |
|  |  | FC | p-value | FC | p-value | FC | p-value |
| **ABCG2** | ATP binding cassette subfamily G member 2 (Junior blood group) |  |  |  |  | 1.45 | 0.031 |
| **ADGRG6** | **adhesion G protein-coupled receptor G6** |  |  | **2.16** | **0.012** |  |  |
| **AK1** | adenylate kinase 1 |  |  |  |  | 1.77 | 0.025 |
| **AKAP1** | A-kinase anchoring protein 1 |  |  | -1.77 | 0.035 |  |  |
| **ANPEP** | **alanyl aminopeptidase, membrane** |  |  |  |  | **2.14** | **0.036** |
| **AP2B1** | adaptor related protein complex 2 subunit beta 1 |  |  | -1.20 | 0.045 |  |  |
| **ARHGEF5** | Rho guanine nucleotide exchange factor 5 |  |  | -1.73 | 0.002 | -1.50 | 0.011 |
| **ATM** | ATM serine/threonine kinase |  |  |  |  | -1.54 | 0.04 |
| **AURKA** | aurora kinase A |  |  | -1.43 | 0.038 | -1.87 | 0.036 |
| **B2M** | beta-2-microglobulin | 1.26 | 0.042 | 1.41 | 0.031 | 1.44 | 0.008 |
| **BAG1** | **BCL2 associated athanogene 1** |  |  | **-2.13** | **0.046** | -1.79 | 0.031 |
| **BBC3** | **BCL2 binding component 3** |  |  |  |  | **2.85** | **0.031** |
| **BCL2L1** | BCL2 like 1 |  |  |  |  | -1.55 | 0.008 |
| **BIRC5** | **baculoviral IAP repeat containing 5** |  |  |  |  | **-3.83** | **0.006** |
| **BRCA2** | BRCA2, DNA repair associated | -1.95 | 0.049 |  |  |  |  |
| **BUB1** | **BUB1 mitotic checkpoint serine/threonine kinase** | **2.73** | **0.008** |  |  | 1.80 | 0.023 |
| **CA9** | carbonic anhydrase 9 |  |  |  |  | -1.54 | 0.012 |
| **CAPNS1** | calpain small subunit 1 |  |  | -1.25 | 0.039 | -1.27 | 0.049 |
| **CCNB1** | cyclin B1 |  |  | -1.61 | 0.047 | -1.87 | 0.017 |
| **CCND3** | cyclin D3 |  |  |  |  | 1.59 | 0.023 |
| **CCT5** | chaperonin containing TCP1 subunit 5 |  |  |  |  | -1.52 | 0.039 |
| **CD44** | CD44 molecule (Indian blood group) | -1.25 | 0.023 |  |  | -1.46 | 0.006 |
| **CDC20** | cell division cycle 20 |  |  | 1.10 | 0.012 |  |  |
| **CDC25A** | **cell division cycle 25A** |  |  |  |  | **-3.14** | **0.015** |
| **CDC42** | **cell division cycle 42** | **-3.68** | **0.04** |  |  |  |  |
| **CDK13** | **cyclin dependent kinase 13** |  |  |  |  | **-3.79** | **0.044** |
| **CDKN1A** | **cyclin dependent kinase inhibitor 1A** |  |  | **5.41** | **0.001** | **6.42** | **0.0001** |
| **CDKN2D** | **cyclin dependent kinase inhibitor 2D** | 1.84 | 0.035 | **4.51** | **0.001** | **3.77** | **0.007** |
| **CHPT1** | choline phosphotransferase 1 |  |  | -1.40 | 0.029 |  |  |
| **CNBP** | CCHC-type zinc finger nucleic acid binding protein | 1.22 | 0.027 |  |  | 1.21 | 0.041 |
| **COX6C** | cytochrome c oxidase subunit 6C |  |  |  |  | -1.54 | 0.014 |
| **COX7A2** | cytochrome c oxidase subunit 7A2 |  |  |  |  | -1.75 | 0.036 |
| **CRAT** | **carnitine O-acetyltransferase** |  |  | **2.04** | **0.006** | **2.17** | **0.004** |
| **CTNNB1** | catenin beta 1 |  |  |  |  | 1.23 | 0.04 |
| **CTPS1** | **CTP synthase 1** |  |  | -1.70 | 0.025 | **-2.72** | **0.013** |
| **CUL1** | **cullin 1** |  |  | **-2.26** | **0.03** | **-3.20** | **0.015** |
| **DHCR7** | 7-dehydrocholesterol reductase |  |  |  |  | 1.72 | 0.035 |
| **DHRS2** | **dehydrogenase/reductase 2** |  |  | **5.56** | **0.029** | **5.41** | **0.03** |
| **E2F5** | E2F transcription factor 5 |  |  |  |  | -1.34 | 0.041 |
| **EGLN1** | egl-9 family hypoxia inducible factor 1 |  |  | -1.62 | 0.017 | -1.72 | 0.014 |
| **EIF5** | **eukaryotic translation initiation factor 5** |  |  | **-2.23** | **0.032** | **-2.12** | **0.001** |
| **ERCC3** | **ERCC excision repair 3, TFIIH core complex helicase subunit** |  |  |  |  | **-2.34** | **0.042** |
| **ETV3** | **ETS variant 3** |  |  |  |  | **2.09** | **0.044** |
| **ETV6** | ETS variant 6 |  |  | 1.38 | 0.031 |  |  |
| **EXT1** | exostosin glycosyltransferase 1 |  |  | 1.85 | 0.037 | **2.57** | **0.002** |
| **EZH2** | enhancer of zeste 2 polycomb repressive complex 2 subunit |  |  |  |  | -1.44 | 0.045 |
| **EZR** | ezrin |  |  |  |  | 1.56 | 0.008 |
| **F2R** | **coagulation factor II thrombin receptor** | **-4.37** | **0.038** |  |  | **-9.26** | **0.014** |
| **FBP1** | fructose-bisphosphatase 1 |  |  |  |  | -1.28 | 0.027 |
| **FOS** | **Fos proto-oncogene, AP-1 transcription factor subunit** |  |  | 1.92 | 0.019 | **2.17** | **0.011** |
| **FOSL2** | FOS like 2, AP-1 transcription factor subunit |  |  |  |  | 1.72 | 0.019 |
| **FZD5** | **frizzled class receptor 5** |  |  | **2.23** | **0.024** | **2.44** | **0.021** |
| **FZD9** | **frizzled class receptor 9** |  |  | **-18.18** | **0.023** |  |  |
| **GBE1** | 1,4-alpha-glucan branching enzyme 1 |  |  |  |  | -1.87 | 0.003 |
| **GCN1** | GCN1, eIF2 alpha kinase activator homolog |  |  | -1.29 | 0.041 | -1.59 | 0.023 |
| **GDF15** | **growth differentiation factor 15** |  |  | **2.12** | **0.01** | **2.39** | **0.017** |
| **GNAZ** | **G protein subunit alpha z** |  |  | **3.01** | **0.006** | **3.18** | **0.01** |
| **GPR39** | G protein-coupled receptor 39 |  |  | -1.79 | 0.044 |  |  |
| **GRB7** | growth factor receptor bound protein 7 |  |  |  |  | 1.61 | 0.033 |
| **GTF2I** | general transcription factor IIi |  |  |  |  | -1.79 | 0.012 |
| **HMBS** | hydroxymethylbilane synthase | -1.39 | 0.003 | -1.72 | 0.019 |  |  |
| **HSPA8** | heat shock protein family A (Hsp70) member 8 |  |  |  |  | -1.54 | 0.036 |
| **HSPB1** | **heat shock protein family B (small) member 1** |  |  | 1.70 | 0.02 | **2.02** | **0.008** |
| **HYAL1** | **hyaluronoglucosaminidase 1** |  |  | **3.38** | **0.011** | **7.09** | **0.004** |
| **ICAM1** | **intercellular adhesion molecule 1** |  |  | **3.23** | **0.041** | **6.42** | **0.006** |
| **IDUA** | **iduronidase, alpha-L-** |  |  | **2.02** | **0.02** |  |  |
| **IER3** | **immediate early response 3** |  |  | 1.91 | 0.01 | **2.58** | **0.003** |
| **IFNGR1** | interferon gamma receptor 1 |  |  | 1.59 | 0.025 | 1.89 | 0.012 |
| **IGF2R** | insulin like growth factor 2 receptor | -1.34 | 0.024 |  |  |  |  |
| **INSR** | insulin receptor | -1.50 | 0.042 |  |  |  |  |
| **IP6K2** | inositol hexakisphosphate kinase 2 |  |  |  |  | -1.31 | 0.037 |
| **ITGA3** | integrin subunit alpha 3 |  |  |  |  | 1.24 | 0.03 |
| **JUN** | **Jun proto-oncogene, AP-1 transcription factor subunit** |  |  | **2.16** | **0.005** | **2.79** | **0.003** |
| **LAMB1** | **laminin subunit beta 1** |  |  | **2.29** | **0.047** | **3.01** | **0.037** |
| **LAMP2** | lysosomal associated membrane protein 2 |  |  | 1.29 | 0.048 |  |  |
| **LCN2** | lipocalin 2 |  |  |  |  | 1.85 | 0.043 |
| **LITAF** | **lipopolysaccharide induced TNF factor** |  |  |  |  | **-3.36** | **0.049** |
| **LRPAP1** | **LDL receptor related protein associated protein 1** |  |  | **-2.02** | **0.031** |  |  |
| **M6PR** | mannose-6-phosphate receptor, cation dependent |  |  | -1.39 | 0.031 |  |  |
| **MAD2L1** | mitotic arrest deficient 2 like 1 |  |  |  |  | -1.75 | 0.024 |
| **MAPK12** | **mitogen-activated protein kinase 12** |  |  | 1.57 | 0.025 | **2.65** | **0.004** |
| **MAPK8** | **mitogen-activated protein kinase 8** |  |  | **3.58** | **0.033** | **2.64** | **0.035** |
| **MCM2** | **minichromosome maintenance complex component 2** |  |  | -1.83 | 0.025 | **-2.80** | **0.012** |
| **MCM6** | **minichromosome maintenance complex component 6** |  |  | -1.76 | 0.013 | **-2.08** | **0.01** |
| **MDM2** | MDM2 proto-oncogene |  |  |  |  | -1.72 | 0.044 |
| **MKI67** | marker of proliferation Ki-67 |  |  |  |  | -1.82 | 0.027 |
| **MYC** | **MYC proto-oncogene, bHLH transcription factor** |  |  |  |  | **-4.67** | **0.002** |
| **MYCN** | **MYCN proto-oncogene, bHLH transcription factor** | **12.65** | **0.029** |  |  |  |  |
| **NDRG1** | N-myc downstream regulated 1 |  |  | 1.87 | 0.03 | 1.86 | 0.034 |
| **NFKB2** | **nuclear factor kappa B subunit 2** |  |  | **2.57** | **0.015** | **2.72** | **0.015** |
| **NR1D1** | **nuclear receptor subfamily 1 group D member 1** |  |  | **2.11** | **0.031** | 1.80 | 0.042 |
| **NR4A1** | **nuclear receptor subfamily 4 group A member 1** |  |  |  |  | **6.41** | **0.031** |
| **PAK1** | p21 (RAC1) activated kinase 1 |  |  | 1.59 | 0.014 |  |  |
| **PCNA** | proliferating cell nuclear antigen |  |  | -1.56 | 0.005 | -1.54 | 0.008 |
| **PEA15** | **proliferation and apoptosis adaptor protein 15** |  |  | 1.90 | 0.011 | **2.17** | **0.006** |
| **PHB** | prohibitin |  |  |  |  | -1.91 | 0.009 |
| **PIK3CG** | phosphatidylinositol-4,5-bisphosphate 3-kinase catalytic subunit gamma | 1.23 | 0.014 |  |  |  |  |
| **PLCB1** | phospholipase C beta 1 |  |  |  |  | -1.21 | 0.034 |
| **PLK2** | polo like kinase 2 |  |  | 1.40 | 0.01 | 1.30 | 0.022 |
| **PPARG** | peroxisome proliferator activated receptor gamma |  |  |  |  | -1.70 | 0.025 |
| **PRKCA** | **protein kinase C alpha** |  |  | 1.64 | 0.016 | **2.11** | **0.006** |
| **PRKCD** | **protein kinase C delta** |  |  | **2.47** | **0.011** | **2.56** | **0.008** |
| **PRKCZ** | protein kinase C zeta |  |  | 1.56 | 0.026 |  |  |
| **PRNP** | **prion protein** |  |  | -1.72 | 0.038 | **-3.52** | **0.006** |
| **RAB6B** | **RAB6B, member RAS oncogene family** |  |  | **8.23** | **0.001** | **6.39** | **0.012** |
| **RAC1** | Rac family small GTPase 1 | 1.87 | 0.02 | 1.92 | 0.032 | 1.92 | 0.017 |
| **RAC2** | **Rac family small GTPase 2** |  |  | **2.28** | **0.035** | **2.90** | **0.024** |
| **RALBP1** | **ralA binding protein 1** |  |  |  |  | **-3.52** | **0.006** |
| **RBL2** | **RB transcriptional corepressor like 2** |  |  |  |  | **2.66** | **0.027** |
| **RHOB** | **ras homolog family member B** |  |  | **2.25** | **0.008** | **3.40** | **0.001** |
| **RHOD** | ras homolog family member D |  |  |  |  | 1.78 | 0.04 |
| **RPN2** | ribophorin II |  |  | -1.23 | 0.035 | -1.50 | 0.004 |
| **RRM2** | **ribonucleotide reductase regulatory subunit M2** |  |  |  |  | **-2.71** | **0.009** |
| **SEMA4D** | semaphorin 4D |  |  | 1.96 | 0.01 | 1.55 | 0.034 |
| **SEPT9** | **septin 9** |  |  |  |  | **6.92** | **0.009** |
| **SERPINH1** | **serpin family H member 1** | 1.21 | 0.049 |  |  | **-3.75** | **0.018** |
| **SFN** | **stratifin** |  |  |  |  | **-2.06** | **0.002** |
| **SFPQ** | **splicing factor proline and glutamine rich** |  |  |  |  | **-2.42** | **0.035** |
| **SHB** | **SH2 domain containing adaptor protein B** |  |  | **2.03** | **0.024** | 1.90 | 0.034 |
| **SLC20A1** | **solute carrier family 20 member 1** |  |  | 1.60 | 0.027 | **2.54** | **0.01** |
| **SLC7A1** | solute carrier family 7 member 1 |  |  |  |  | -1.50 | 0.007 |
| **SRSF7** | **serine and arginine rich splicing factor 7** |  |  |  |  | **-2.08** | **0.045** |
| **STAT2** | signal transducer and activator of transcription 2 |  |  | 1.54 | 0.016 |  |  |
| **TBX3** | T-box 3 |  |  | 1.56 | 0.033 |  |  |
| **TFRC** | transferrin receptor |  |  |  |  | -1.62 | 0.003 |
| **TGFBR3** | **transforming growth factor beta receptor 3** | **2.40** | **0.021** |  |  |  |  |
| **TNFRSF10A** | TNF receptor superfamily member 10a |  |  |  |  | -1.59 | 0.036 |
| **TNFRSF10B** | **TNF receptor superfamily member 10b** |  |  |  |  | **2.11** | **0.004** |
| **TNFRSF1A** | TNF receptor superfamily member 1A | 1.12 | 0.029 |  |  |  |  |
| **TNK2** | **tyrosine kinase non receptor 2** |  |  |  |  | **2.26** | **0.033** |
| **TOB1** | transducer of ERBB2, 1 |  |  | 1.57 | 0.048 | 1.56 | 0.015 |
| **TP53** | **tumor protein p53** |  |  |  |  | **-2.28** | **0.002** |
| **TRADD** | TNFRSF1A associated via death domain |  |  |  |  | -1.70 | 0.028 |
| **TRAM1** | translocation associated membrane protein 1 |  |  |  |  | -1.53 | 0.02 |
| **TRIP13** | thyroid hormone receptor interactor 13 |  |  | -1.61 | 0.019 | -1.95 | 0.019 |
| **VEGFA** | vascular endothelial growth factor A |  |  | 1.66 | 0.01 | 1.69 | 0.011 |
| **XRCC6** | X-ray repair cross complementing 6 | 1.27 | 0.038 |  |  |  |  |

**Supplementary Figure 4**. **(A)** IL-8 production from BSCCs with viable *F. nucleatum*, HK *F. nucleatum*, or vehicle-treatment (*, *p* < 0.01, Tukey’s multiple comparisons test). **(B, C, D)** Networks of predicted disease or functional annotation and related differentially expressed genes for (B) BSCCs with HK *F. nucleatum* 23726, (C) BSCCs with *F. nucleatum* 23726, and (D) BSCCs with *F. nucleatum* 25586. Genes that were up-regulated are shown in red and genes that were down-regulated are shown in green. Color intensity represents relative level of up- or down-regulation. Predicted induction of diseases and/or functions are represented in orange and predicted inhibition is represented in blue.


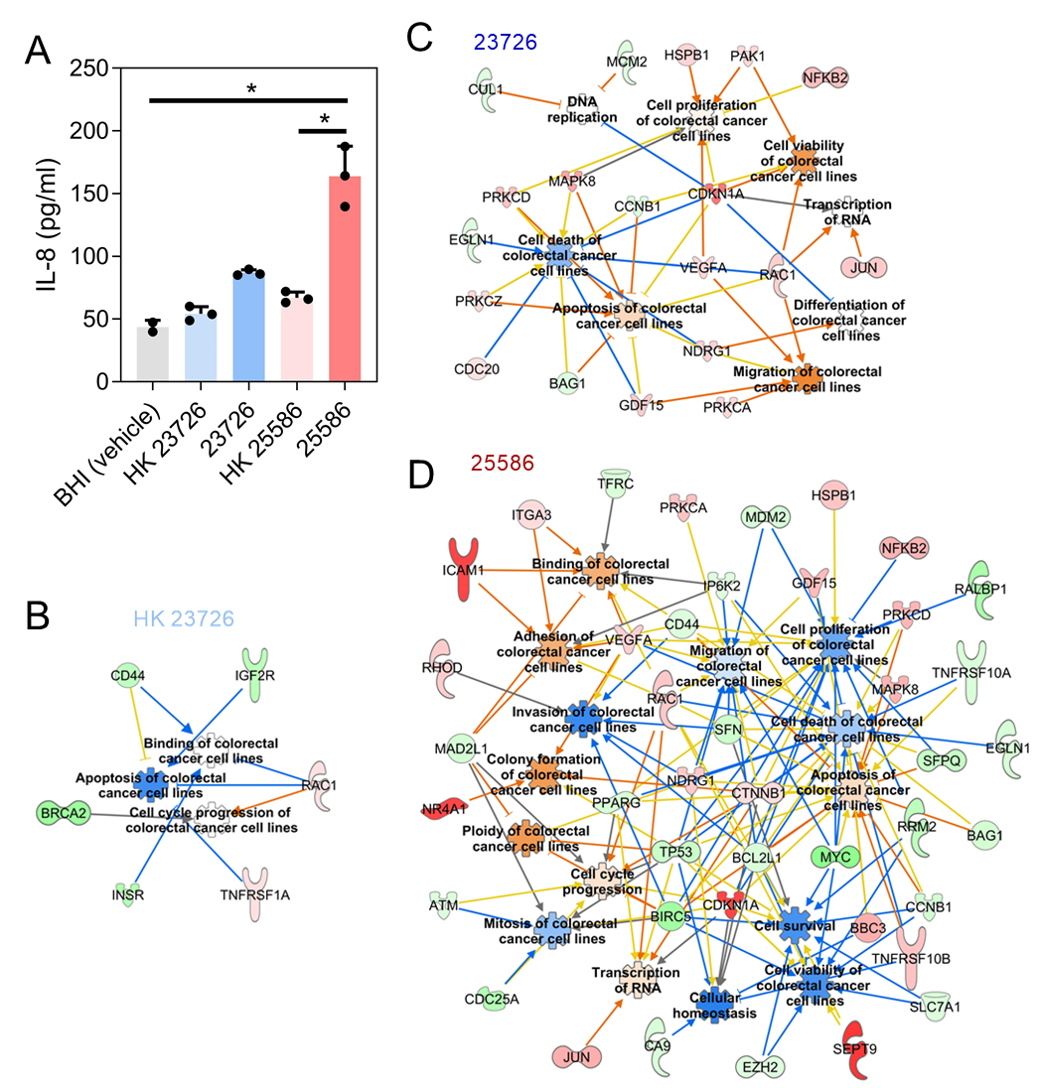


**Supplementary Figure 5**. QQQ-MS data of significantly differential abundant metabolites (*p* < 0.05, one way ANOVA). Data is shown as relative metabolite level to vehicle control at 0 h over time.


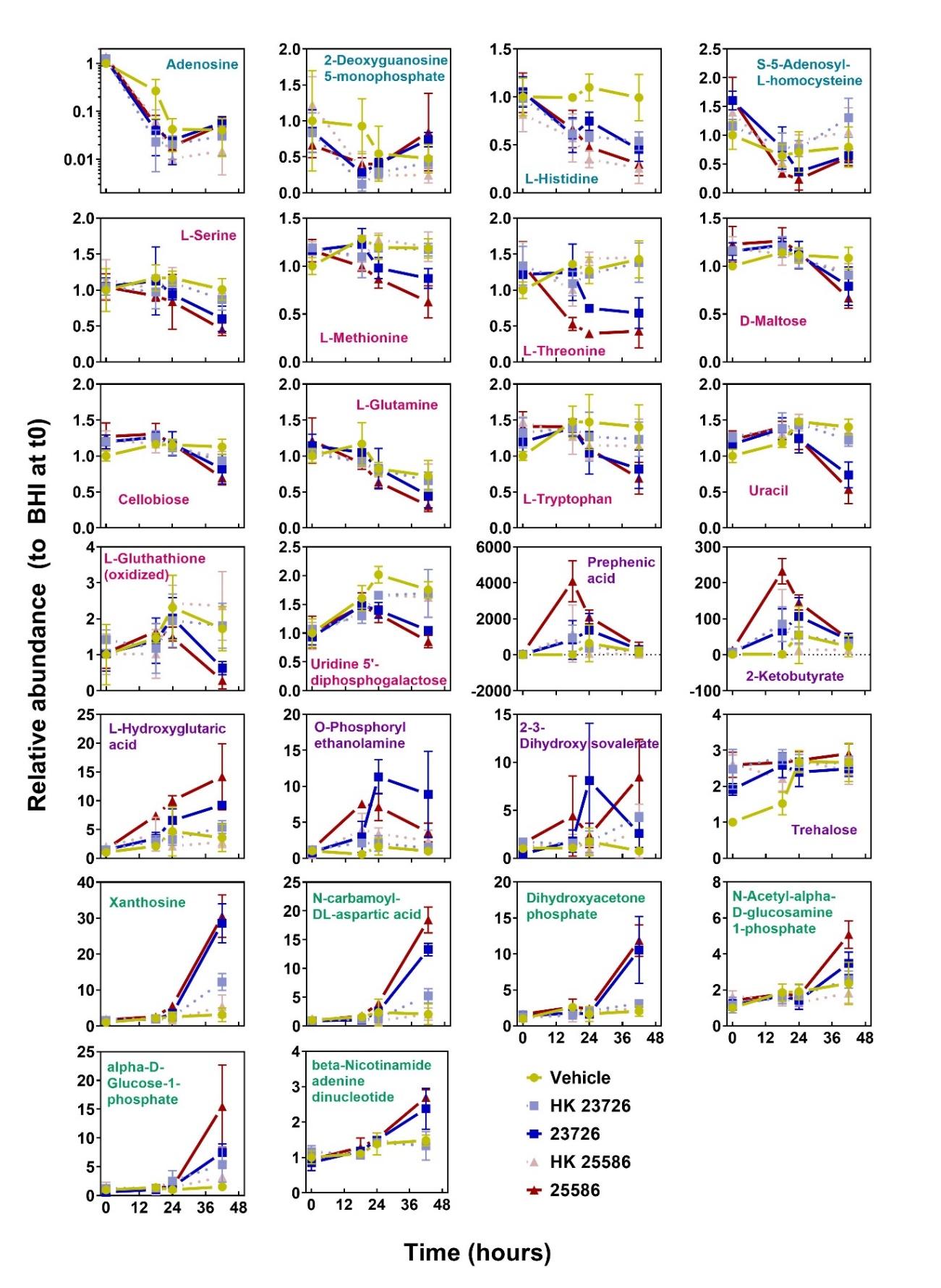


**Supplementary Figure 6**. Colony morphology of diverse *Fusobacteria* in ULA microplates with McCoy media grown under anaerobic conditions (without tumor spheroid present). Scale bar represents 500 μm.


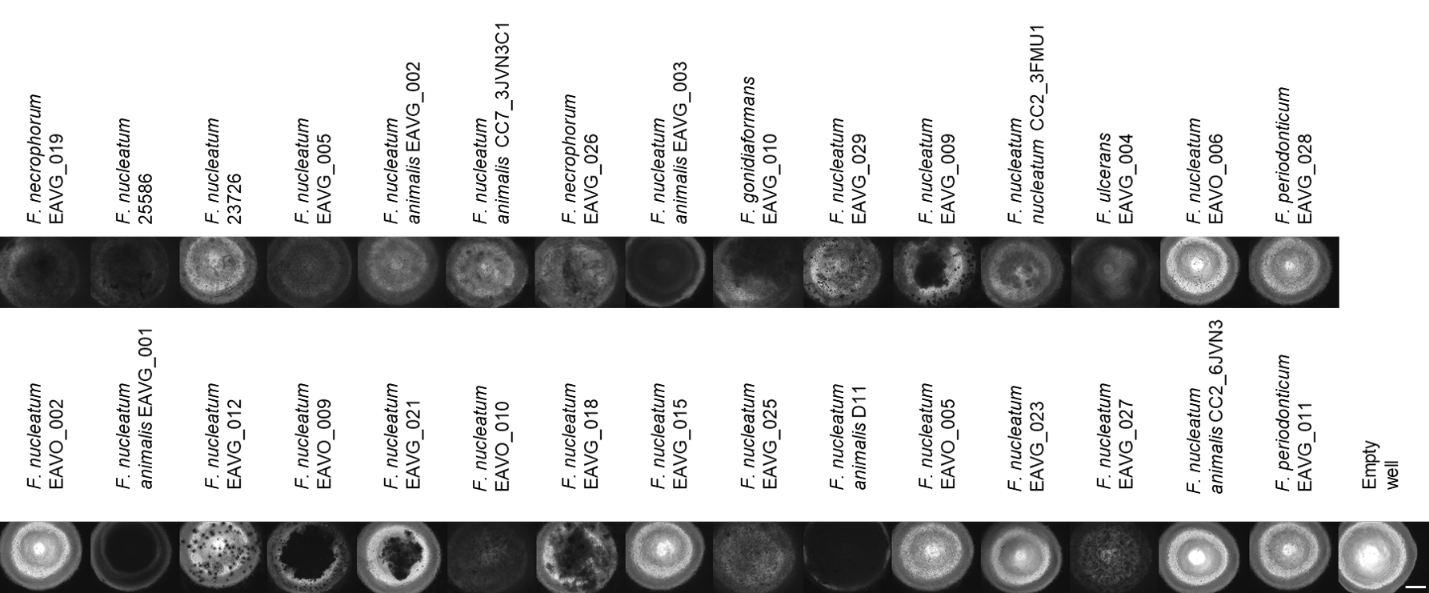


**Supplementary Table 3**. LC/MS method for targeted analysis of central carbon metabolites

| LC Conditions | |
| --- | --- |
| Column | Agilent ZORBAX RRHD Extend-C18, 2.1 × 150 mm, 1.8 um (Part # 759700-902) |
| Guard column | ZORBAX Eclipse Plus C18, 2.1 mm, 1.8 µm, UHPLC guard column |
| Column temperature | 40 ⁰C |
| Needle wash | Methanol:water (50:50) with 15 mM glacial acetic acid |
| Mobile phase | (A) Water:Methanol (97:3) with 15 mM glacial acetic acid and 10 mM tributylamine (Acros Organics AC139320010)  (B) Methanol with 15 mM glacial acetic acid and 10 mM tributylamine  (D) Acetonitrile |
| Flow rate | Variable (see gradient table) |
| Gradient program | | Time (min) | % A | % B | %D | Flow (mL/min) | | | --- | --- | --- | --- | --- | --- | | 2.5 | 100 | 0 | 0 | | 0.25 | | 7.5 | 80 | 20 | 0 | | 0.25 | | 13.00 | 55 | 45 | 0 | | 0.25 | | 20.00 | 1 | 99 | 0 | | 0.25 | | 24.00 | 1 | 99 | 0 | | 0.25 | | 24.05 | 1 | 0 | 99 | | 0.25 | | 27.00 | 1 | 0 | 99 | | 0.25 | | 27.50 | 1 | 0 | 99 | | 0.8 | | 31.35 | 1 | 0 | 99 | | 0.8 | | 31.50 | 1 | 0 | 99 | | 0.6 | | 32.25 | 100 | 0 | 0 | | 0.4 | | 39.90 | 100 | 0 | 0 | | 0.4 | | 40.00 | 100 | 0 | 0 | | 0.25 | |
| Stop time | 40 mins |
| MS Ionization mode | ESI negative |

**Supplementary Table 4**. LC/MS method for untargeted analysis

| LC Conditions | |
| --- | --- |
| Column | Agilent InfinityLab Poroshell 120 HILIC-Z, 2.1 x 100 mm, 2.7 μm (p/n 685775-924) |
| Guard column | Agilent InfinityLab Poroshell 120 HILIC-Z, 4.6 x 5 mm, 2.7 μm (p/n 820750-933) |
| Column temperature | 30 ⁰C |
| Mobile phase | (A) 200 mM ammonium formate (pH 3): H2O (10:90)  (B) 90:10, ACN:H2O with 200 mM ammonium formate, pH 3 + 5 µM medronic acid |
| Flow rate | 0.5 mL/min |
| Gradient program | | Time (min) | % A | % B | | --- | --- | --- | | 0.0 | 0 | 100 | | 11.50 | 30 | 70 | | 13.50 | 60 | 40 | | 14.50 | 60 | 40 | | 15.00 | 0 | 100 | |
| Stop time | 20 mins |

**Supplementary Table 5.** Metabolites identified by LC/MS QQQ analysis

| **Compound name** | **CAS ID** | **Compound name** | **CAS ID** |
| --- | --- | --- | --- |
| 2-2-Dimethyl Succinic acid | 597-43-3 | D-Glucose 6-phosphate | 54010-71-8 |
| 2-3-Dihydroxybenzoic acid | 303-38-8 | Dihydroxyacetone phosphate | 57-04-5 |
| 2-3-Dihydroxyisovalerate | 1756-18-9 | DL-2-Aminoadipic acid | 542-32-5 |
| 2-3-Pyridinedicarboxylic acid | 89-00-9 | DL-Glyceraldehyde 3-phosphate | 591-59-3 |
| 2-Deoxyadenosine | 16373-93-6 | DL-Isocitric acid | 320-77-4 |
| 2-Deoxycytidine | 951-77-9 | D-Maltose | 69-79-4 |
| 2-Deoxycytidine 5-monophosphate | 1032-65-1 | D-Mannose | 31103-86-3 |
| 2-Deoxyguanosine | 961-07-9 | D-pantothenic acid | 79-83-4 |
| 2-Deoxyguanosine 5-monophosphate | 902-04-5 | D-Ribose 5-phosphate | 4300-28-1 |
| 2-Deoxyinosine | 890-38-0 | D-Ribulose 1,5-biphosphate | 2002-28-0 |
| 2-Deoxyuridine | 951-78-0 | D-Sedoheptulose-7-phosphate | 2646-35-7 |
| 2-Isopropylmalic acid | 3237-44-3 | D-Xylose | 58-86-6 |
| 2-Ketobutyrate | 600-18-0 | D-Xylulose-5-phosphate | 60802-29-1 |
| 2-Methyl-1-butanol | 1565-80-6 | Epicatechin | 490-46-0 |
| 3-Hydroxyanthranilic acid | 548-93-6 | Flavin adenine dinucleotide | 146-14-5 |
| 3-Indoleacetic acid | 87-51-4 | Galactonic acid | 576-36-3 |
| 3-Methylglutaric acid | 626-51-7 | Glyceric acid | 473-81-4 |
| 4-Aminobenzoic acid | 150-13-0 | Guanine | 73-40-5 |
| 4-Guanidobutyric acid | 463-00-3 | Guanosine | 118-00-3 |
| 4-Hydroxybenzoic acid | 99-96-7 | Guanosine 3,5-cyclic monophosphate | 7665-99-8 |
| 4-Hydroxy-L-glutamic acid | 2485-33-8 | Hypoxanthine | 68-94-0 |
| 4-Hydroxyphenyl-pyruvic acid | 156-39-8 | Inosine | 58-63-9 |
| 4-Methyl-2-oxovaleric acid | 816-66-0 | Inosine 5-monophosphate | 86-04-4 |
| 4-Pyridoxic acid | 82-82-6 | Isopentyl acetate | 123-92-2 |
| 4-quinolinol | 611-36-9 | Itaconic acid | 97-65-4 |
| 5-Deoxy-5-(methylthio)adenosine | 2457-80-9 | Ketoisovaleric acid | 759-05-7 |
| 6-Hydroxynicotinic acid | 5006-66-6 | Ketovaleric acid | 1821-02-9 |
| Adenine | 73-24-5 | Lactic acid | 50-21-5 |
| Adenosine | 58-61-7 | L-Arabinose | 5328-37-0 |
| Adenosine 3-5-cyclic monophosphate | 60-92-4 | L-Arabitol | 7643-75-6 |
| Adenosine 5-diphosphate | 58-64-0 | L-Arginine | 74-79-3 |
| Adenosine 5-monophosphate | 61-19-8 | L-asparagine | 70-47-3 |
| Adipic acid | 124-04-9 | L-Aspartic Acid | 56-84-8 |
| AICAR | 2627-69-2 | L-Citrulline | 372-75-8 |
| Allantoin | 97-59-6 | L-Cystine | 56-89-3 |
| alpha-D(+)Mannose 1-phosphate | 27251-84-9 | L-Dihydroorotic acid | 5988-19-2 |
| alpha-D-Glucose-1-phosphate | 59-56-3 | L-Glutamic acid | 56-86-0 |
| alpha-Ketoglutaric acid | 328-50-7 | L-Glutamine | 56-85-9 |
| Arabinose-5-phosphate | 13137-52-5 | L-Gluthathione (oxidized) | 27025-41-8 |
| beta-Nicotinamide adenine dinucleotide | 53-84-9 | L-Histidine | 71-00-1 |
| beta-Nicotinamide mononucleotide | 1094-61-7 | L-Hydroxyglutaric acid | 13095-48-2 |
| Cellobiose | 528-50-7 | L-Isoleucine | 61-90-5 |
| Citramalic acid | 2306-22-1 | L-Kynurenine | 2922-83-0 |
| Citric acid | 77-92-9 | L-Leucine | 61-90-5 |
| Creatine | 57-00-1 | L-Malic acid | 97-67-6 |
| Creatinine | 60-27-5 | L-Methionine | 63-68-3 |
| Cytidine | 65-46-3 | L-Phenylalanine | 63-91-2 |
| Cytidine-5-monophosphate | 63-37-6 | L-Proline | 147-85-3 |
| D-erythro-Dihydrosphingosine | 764-22-7 | L-Serine | 56-45-1 |
| D-Fructose 1,6-biphosphate | 488-69-7 | L-Sorbose | 87-79-6 |
| D-Fructose 6-phosphate | 643-13-0 | L-Threonine | 72-19-5 |
| D-Gluconic acid | 526-95-4 | L-Tryptophan | 73-22-3 |
| L-Tyrosine | 60-18-4 | Quinic acid | 77-95-2 |
| Maleic acid | 110-16-7 | Riboflavin | 83-88-5 |
| Malonic acid | 141-82-2 | Ribonic acid gamma lactone | 5336-08-3 |
| Melibiose | 5340-95-4 | S-5-Adenosyl-L-homocysteine | 979-92-0 |
| Mevalonic acid | 150-97-0 | Salicylic acid | 69-72-7 |
| m-Hydroxybenzoic acid | 99-06-9 | Succinic acid | 110-15-6 |
| myo-Inositol | 87-89-8 | Succinic semialdehyde | 692-29-5 |
| N-Acetyl-alpha-D-glucosamine 1-phosphate | 28446-21-1 | Taurine | 107-35-7 |
| N-Acetyl-D-glucosamine 6-phosphate | 1746-32-3 | Taurocholic acid | 83830-80-2 |
| N-Acetylglutamic acid | 5817-08-3 | Thiamine | 67-03-8 |
| N-Acetylneuraminic acid | 131-48-6 | Thymidine | 50-89-5 |
| N-carbamoyl-DL-aspartic acid | 923-37-5 | Thymine | 65-71-4 |
| N-Carbamyl-L-glutamic acid | 1188-38-1 | trans-4-Hydroxy-L-proline | 51-35-4 |
| N-Formyl-L-Tyrosine | 13200-86-7 | Trehalose | 99-20-7 |
| Nicotinic acid | 59-67-6 | Trehalose 6-phosphate | 4484-88-2 |
| o-Hydroxy hippuric acid | 487-54-7 | Uracil | 66-22-8 |
| O-Phosphorylethanolamine | 1071-23-4 | Uric acid | 69-93-2 |
| Orotic acid | 65-86-1 | Uridine | 58-96-8 |
| O-Succinyl-L-homoserine | 1492-23-5 | Uridine 5'-diphosphogalactose | 2956-16-3 |
| Oxamic acid | 471-47-6 | Uridine 5-diphosphoglucose | 133-89-1 |
| Phenylpyruvic acid | 156-06-9 | Uridine 5-monophosphate | 58-97-9 |
| Prephenic acid | 129-49-8 | Vanillic acid | 121-34-6 |
| Pyridoxal 5 phosphate | 54-47-7 | Xanthine | 69-89-6 |
| Pyridoxal hydrochloride | 65-22-5 | Xanthosine | 146-80-5 |
| Pyridoxine | 65-23-6 | Xylitol | 87-99-0 |
| Pyruvic acid | 127-17-3 |  |  |

***alpha-D(+)Mannose 1-phosphate could not be differentiated from alpha-D-Glucose-1-phosphate by LC/MS QQQ analysis**
